# Bioactive profiling and evaluation of anti-proliferative and anti-cancerous properties of Shivagutika, an Indian polyherbal formulation synchronizing *in vitro* and *in silico* approaches

**DOI:** 10.3389/fchem.2023.1195209

**Published:** 2023-05-17

**Authors:** Pushpa V. H., Mahadevaswamy G. Kuruburu, Jayanthi M. K., Akshaya Simha N., Abdullatif Taha Babakr, Rajesh Sreenivasan, Ramith Ramu, SubbaRao V. Madhunapantula

**Affiliations:** ^1^ Department of Pharmacology, JSS Medical College, JSS Academy of Higher Education and Research, Mysuru, Karnataka, India; ^2^ Center of Excellence in Molecular Biology and Regenerative Medicine (A DST-FIST Supported Center), Department of Biochemistry (A DST-FIST Supported Department), JSS Medical College, JSS Academy of Higher Education and Research, Mysuru, Karnataka, India; ^3^ Department of Biotechnology and Bioinformatics, JSS Academy of Higher Education and Research, Mysuru, Karnataka, India; ^4^ Department of Medical Biochemistry, College of Medicine, Umm Al-Qura University, Makkah, Saudi Arabia; ^5^ The Sadvaidyasala Private Limited, Nanjangud, Karnataka, India; ^6^ Special Interest Group in Cancer Biology and Cancer Stem Cells (SIG-CBCSC), JSS Medical College, JSS Academy of Higher Education and Research, Mysuru, Karnataka, India

**Keywords:** Shivagutika, caspase 3, Sciadopitysin, breast cancer, molecular modelling

## Abstract

Shivagutika is a polyherbal formulation mentioned in Ayurveda, the oldest system of medicine. The aim of this study was to investigate the anti-breast cancer potential of DCM extract of Shivagutika using MCF-7, MDA-MB-231, and MDA-MB-468. Primarily, various extracts of Shivagutika were prepared and subjected to primary *in vitro* analysis—total protein, phenolic acid content, and flavonoid content. DCM extract among all the extracts showed the promising results hence, it was subjected to LC-MS/MS analysis to identify the phytochemicals. The same extract was subjected to anti-proliferation assay and anti-cancer assay. It inhibited all the 3 cell lines and increased the activity of Caspase 3, pro-apoptotic protein. Further, to find the potent molecule(s) *in silico* analysis (molecular docking and molecular dynamics simulation studies) was performed. Sciadopitysin was identified as a potent molecule among all phytochemicals as it interacted with Caspase 3 with a binding energy of −7.2 kcal/mol. MD simulation studies also revealed that Sciadopitysin was stable inside the binding pocket of Caspase 3 by interacting with the amino acids in the catalytic site thereby activating the Caspase 3 levels. By all the above results, Shivagutika could be used as a potent anti-breast cancer agent (specifically DCM extract of Shivagutika) which could decrease the cases of breast cancer in future.

## Introduction

Breast cancer is the second-leading cause of mortality and the most prevalent kind of cancer. It is the second highest cause of cancer-related death in women between the ages of 45 and 55 (Jemal et al., 2009) and the main cause of death among them. Most of the time, full tissue excision, chemotherapy, radiation, and hormone therapy are required for the treatment of breast cancer, which affects almost one in eight women (Ataollahi et al., 2015). Breast cancer is a kind of tissue cancer that mostly affects ducts, which are small tubes that convey milk, and the inner layer of milk glands or lobules (Sariego et al., 1995). Age (Steiner and Klubert, 2008), high hormone levels (Yager et al., 2006), race, economic standing, and iodine deficiency in the diet (Venturi, 2001; Aceves et al., 2005; Ii et al., 2008) are the main risk factors for cancer. Viruses have a part to play in one of the stages of the multi-stage illness breast cancer.

Particularly for anticancer and anti-infectious medicines, natural products have become a standard in the discovery and development of innovative pharmaceuticals (Newman and Cragg, 2020). It is interesting that around 50% of anti-cancer medications and treatments are either made from natural substances or natural products (Christensen, 2021). Alkaloids, polyphenols, flavonoids, terpenoids, and polysaccharides are only a few of the herbal ingredients being utilised to treat communicable illnesses and different malignancies (Sofowora et al., 2013; Nwodo et al., 2016). Modulation of the immune system through phytochemicals could be a mode of therapy to cure diseases (Amin et al., 2019). Although there are several therapy options and interventions for these diseases, most of them continue to be incurable due to medication resistance. These phytochemicals can be delivered by combining with materials such as poly (glycolide-co-trimethylene carbonate-co-ε-caprolactone) which protects the phytochemical from the external agents after it qualifies the necessary tests on animals (Ali and Amin, 2022). Nevertheless, some natural products do have adverse effects. To enhance the effectiveness and results of plant-based medicinal agents, new plant-derived compounds must be sought for and discovered.

According to mythology, Lord Shiva gave his son Lord Ganesh the remedy known as Shiva Gutika to treat Premeha, a disease characterised by clinical disorders including diabetes, metabolic syndrome, and obesity. Other than this, there has not been any mention of the traditional applications for this blend of herbs. Shilajathu is the primary ingredient in the Shiva Gutika, without which it is claimed that the immune system will not be enhanced by reducing the three humours, which include health anomalies relating to air and aether (Vata), fire (Pitta), and water (Kapha) (abnormalities) (Pushpa et al., 2021). These phytochemicals have been blended into a single polyherbal preparation from several distinct medicinal plants. Using oral medication, dietary supplements, and immunity-boosting herbs, this polyherbal mix can treat the morbid factor (Ojas). In addition to these roles, it also functions as an antibacterial, antidiabetic, anti-venom, anti-hypolipidemic, anti-inflammatory, analgesic, immuno-modulatory, antiviral, antimicrobial, and antioxidant agent (Bhat, 2014). Despite significant pharmaceutical improvement of uncommon and harmful abnormalities like SEDT and HIV-AIDS, Shiva Gutika’s impact on cancer is yet unknown. Chemotherapy, radiation, and surgery are common forms of treatment, although they are not always effective in removing tumours or free of side effects (Hassannia et al., 2020).

The breast’s milk glands or ducts are the only site of primary breast cancer. Yet, primary breast cancer can occasionally metastasize, or spread, to distant organs like the liver (Khan et al., 2022). In the present study three breast cancer cell lines (MCF-7, MDA-MB-231, and MDA-MB-468) have been used to study the anti-cancer potential of Shivagutika. DCM extract of Shivagutika among all the other extracts showed the promising results during the preliminary *in vitro* analysis. Hence, LC-MS characterisation of DCM extract was performed to identify the active components present in the extract. *In vitro* and *in silico* studies suggested that Sciadopitysin of DCM extract bound strongly with Caspase 3. Therefore, the present investigation indicated that DCM extract of Shivagutika could be used as therapeutic anti-breast cancer agent.

## Materials and methods

### Materials

The National Center for Cell Science (NCCS), located in Pune, Maharashtra, India, provided the breast cancer cell lines MCF-7, MDA-MB-231, and MDA-MB-468. Prof. Annapoorni Rangarajan from the Department of Molecular, Reproduction, Development and Genetics at the Indian Institute of Science in Bengaluru, Karnataka, India, provided the triple positive breast cancer cell line BT-474. Dr. Gopinath, M.S. Principal Scientist, Department of Molecular Nutrition, CSIR-CFTRI, Mysore, Karnataka, India, kindly supported by providing the human keratinocyte cell line HaCaT. Cell culture grade Hybrimax DMSO, SRB, DPPH, Trolox, and TPTZ were acquired from Sigma Chemical Company in St. Louis, Missouri, in the United States. The following products were purchased from HiMedia Laboratories in Mumbai, Maharashtra, India: DMEM, FBS, PenStrep, Glutamax, Trypsin, and DPBS. For cell culture, Techno Plastic Products India, Pvt. Ltd. is a company in Bengaluru, Karnataka, India provided plastic ware (T25, T75, 15.0 mL and 50.0 mL conical tubes, disposable pipettes, micropipettes tips) etc. All other reagents, including solvents, salts, acids, and bases, were of Analytical Reagent (AR) grade, purchased from Sisco Research Laboratories Pvt. Ltd. (SRL), Mumbai, Maharashtra, India.

### Methods

#### Preparation and fractionation of Shivagutika extracts

Weighed 100 g of Shivagutika, dried and powdered. The constituents of Shivagutika were initially extracted with n-hexane (1:2 ratio). Further, the extract obtained from n-hexane was concentrated using a rotary flash evaporator, dried by passing N_2_ gas and freeze-dried at −8°C. Further, the residue left after hexane extraction was subjected to DCM (Dichloromethane) extraction, the DCM extract was concentrated using a rotary flash evaporator, dried by passing N_2_ gas and lyophilized at −8°C. The residue left after DCM extraction was re-extracted using ethanol, concentrated, and freeze-dried at −8°C. The residue left after ethanol extraction was finally extracted using water (aqueous extraction) by employing a stirring method.

The residue after ethanol extraction was dissolved in 600 mL water, stirred for 2 h in a 1,000 mL conical flask and centrifuged at 5,000 rpm for 10 min at 4°C. The supernatant was collected in a separate bottle and dried. Water was removed by adding ether (Subba Rao and Muralikrishna, 2002).

#### Estimation of total phenolic acid by Folin—Ciocalteau method

A modified FC technique, as described by Sánchez-Rangel et al. (2013) was employed to determine the total phenol content of the shivagutika extracts (Batch 52, 53, and 54). In short, 70 µL of gallic acid standards (ranging from 2.5 to 40.0 μg/mL) and appropriately diluted Shivagutika extracts were aliquoted into a 96-well plate and incubated for 30 min at 37°C with 70 µL of FC reagent (SRL, Mumbai, India; diluted 1:1 with water) and 60.0 µL of 4% sodium carbonate. The absorbance of the colour produced was read at 765 nm and the standard graph of gallic acid was used to calculate the total phenolic acid concentration (100 mg/mL of various extracts).

#### Estimation of total flavonoids by aluminium chloride method

The total flavonoid content of the sample was calculated using the aluminium chloride colorimetric technique. Quercetin was used to prepare the standard dilution for the total flavonoid determination. The standard quercetin solution was prepared by serially diluting 5 mg of quercetin in 1 mL of methanol (5–200 μg/mL). Separately, 0.6 mL of diluted standard quercetin solution and extracts were combined with 0.6 mL of 2% aluminium chloride. The resulting mixture was then incubated for 60 min at room temperature. Using a variance UV-visible spectrometer, the reaction mixture’s absorbance was measured at 420 nm against a blank (Akbay et al., 2003).

#### Estimation of total protein content by Bradford method

The determination of total protein content in the Shivagutika extracts was performed using the dye-binding technique described by Bradford (1976).

In this experiment, 250.0 µL of Bradford reagent was used to incubate 10.0 µL of BSA standards (50.0 to 500.0 μg/mL) as well as adequately diluted Shivagutika extracts (batch 52, 53, 54, and 57). Within an hour, the generated colour was read using UV-Visible spectrophotometer set to 595 nm. By comparing the absorbance of test samples with that of standards produced using BSA, the concentration of total protein (mg/gram extract) was determined.

#### Determination of the antioxidant potential using ferric reducing antioxidant power (FRAP)

FRAP assay was carried out as described by Benzie and Strain (1999). Experimentally, 25 mL of 300 mM acetate buffer, pH 3.6, 2.5 mL of 20 mM FeCl_3_, and 2.5 mL of 10 mM TPTZ (2,4,6-trypyridyl-s-triazine) were heated at 37°C to prepare the FRAP reagent. A suitable dilution of Shivagutika extracts (3 batches of DCM extracts) (100 to 500 μg/mL by total phenol content) or FeSO_4_ (standard—200–1,600 µM) was added to 190 µL of FRAP reagent, which was then incubated for 30 min in the dark. The test is based on the principle that Fe^3+^—TPTZ complex is reduced by antioxidant phenolic acids into Fe^2+^ form at low pH, producing a strong blue colour. The absorbance was then measured at 593 nm. Data was plotted against the concentration of extract and converted into FRAP units (equivalent quantity of ferrous sulphate).

#### Determination of the antioxidant potential by DPPH (2,2- diphenyl picrylhydrazyl) radical scavenging activity

DPPH radical scavenging activity was measured according to Blois (1958). DPPH is a protonated radical having the characteristic absorbance maxima at 517 nm which decreases with the scavenging of the proton radical by natural plant extracts. In short, 20.0 µL of Shivagutika extracts (4 batches) containing 100–500 μg/mL (by total phenol) were incubated for 30 min in the dark with 200 µL of DPPH solution (40 mg in 100 mL 100% ethanol), and the absorbance was measured at 536 nm. Ascorbic acid (5 to 100 μg/mL) was used to establish the calibration curve. The results were reported as a percentage of free radical scavenging activity (Pisoschi et al., 2009).

OD of DPPH incubated with solvent = [A_0_−A_1_/A_0_] × 100 A_0_ = DPPH radical scavenging activity expressed as a percentage (%). A_1_ = Ascorbic acid or Extract-incubated OD of DPPH.

#### Identification of phytochemical components of Shivagutika fractions by UPLC—QTOF—MS

The phytochemical components of Shivagutika extracts (4 batches) were dissolved in 100% acetonitrile at a concentration of 0.5 mg/mL, the resulting material was centrifuged at 10,000 rpm for 10 min and the supernatant was subjected to UPLC-QTOF-MS analysis, as described by Chanda et al. (2019). In the experiment, phytochemicals were identified using an Agilent 1290 Infinity LC connected to an Agilent 6500 Quadrupole Time-of-Flight (QTOF) system using Agilent Jet Stream Thermal Gradient Technology. The gradient flow was 5 mM Ammonium Formate in water with 0.05% formic acid (A) and 100% Acetonitrile with 0.05% formic acid (B) with the flow-rate: 0.4 mL/min. Agilent’s PCDL (Personal Compound Database and Library) was mined from the phenol explore database to identify the compounds based on high-resolution, accurate mass data (Du et al., 2012).

#### Determination of anti-proliferative potential

Shivagutika DCM extract’s anti-proliferative property was assessed using the procedure described by Madhunapantula and Robertson (2008). Breast cancer (MCF-7, MDA-MB-231 and MDA-MB-468) were trypsinized and plated at a density of 1,104 cells per well in a 100-L volume in 96 well plates. In a CO_2_ incubator, the cells were allowed to develop until they reached 60%–70% confluence. At this stage, cells were exposed to DCM Shivagutika extract at progressively higher concentrations (62.5 to 1,000 μg/mL) for 48 h, and the number of viable cells was assessed using the sulforhodamine-B (SRB) test (Skehan et al., 1990). In the experimental setup, cells were fixed in a quarter volume of cold, 50% (w/v) TCA, and the plates were maintained at 4°C. The wells are cleansed with water (200 μL X 4 times) to remove serum proteins and TCA after the medium has been in place for 1 h. To stain the cellular proteins, the plates were dried and exposed to 100 µL of 0.4% SRB for 30 min. To get rid of the unbound SRB, the plates were immediately rinsed four times with 200 L of 1% acetic acid. With a PerkinElmer plate reader, the absorbance at 490 nm was measured after the bound SRB was solubilized in 10 mM Tris base solution (100 µL/well). By contrasting the OD values with the control DMSO, the percentage of cell growth inhibition was determined.

#### Anti-cancer activity of soluble Shivagutika DCM extracts

The Shivagutika’s DCM extract were applied to the cell lines for 48 h in order to clarify the mechanism of cell death. Caspase 3 activation is a sign that mammalian cells are beginning to undergo apoptosis. In order to gauge caspase 3 activity, the caspase 3/CPP32 colorimetric test kit was applied. 0.5 × 10^6^ cells were subjected to a 48-h treatment period of the DCM extract of the Shivagutika and the positive control drug ixabepilone (50 mM). The vehicle control was cells treated with 1% DMSO. The kit’s cell lysis buffer was used to gather the cell lysates. Using the BCA technique, protein estimation was performed on the obtained cells. 100 μg of total protein diluted in 50 µL of cell lysis buffer will be used to conduct the caspase 3 test. 50 µL of 2X reaction buffer containing 10 mM DTT and 5 µL of 4 mM DEVD-pNA substrate (200 µM final concentration) were incubated at 37°C for 3 h. Using a PerkinElmer multimode plate reader, the generated yellow hue was read at 405 nm (Du et al., 1997).

#### Cell death assay with acridine orange (AO) and ethidium bromide (EtBr) staining

Acridine Orange (AO) and ethidium bromide cell staining was used to measure cell death (EtBr). MCF-7 and MDAMB-468 cells (0.3 × 10^6^ cell/well in 2 mL medium) were cultured in 6-well plates for about 36 h before being exposed to 100, 250, and 500 µM (total phenol concentration) or vehicle (controls) for 48 h. 100 M of ixabepilone was used as a positive control. Apoptosis test was used as a specific technique as described by Bovilla et al. (2021).

#### Molecular docking simulation

The X-Ray crystallographic structure of Caspase 3 (PDB ID: 2XYP) was obtained from the RCSB PDB database (https://www.rcsb.org) (Accessed on March 2023). The protein molecule was prepared according to Patil et al. (2021b) and Martiz et al. (2022a) using Auto dock Tools 1.5.7. Initially, water and heteroatoms were removed. Further, polar hydrogens were added to stabilize the protein structure. The energy of the protein structure was reduced by using Kollmann-united charges and Gasteiger charges. After minimizing the energy, all atoms were assigned an Auto Dock 4 (AD4) atom type before obtaining the prepared protein structure in PDBQT format for molecular docking simulation. Further, the binding site prediction was done according to Patil et al. (2021b). The grid box measuring 40 Å × 40 Å × 40 Å containing the binding pocket and the attributes of the active site was positioned at x = 37.279455 Å, y = 38.593682 Å, and z = 31.568818 Å was created. During ligand preparation, phytochemical structures were prepared for the molecular docking simulation using Auto Dock Tools 1.5.7 according to Patil et al. (2021b) where the 3D SDF structures were obtained from PubChem database (https://pubchem.ncbi.nlm.nih.gov/) and converted to PDBQT format, and Kollmann-united charges and Gasteiger charges were added to minimize the energy. After energy minimization, ligand molecules were saved in PDBQT format in the same directory as the protein molecule to carry out the docking simulation (Patil et al., 2022a; Martiz et al., 2022c). The virtual screening of the compounds was completed with a command-line-based software known as Auto Dock Vina 1.1.2 which uses the Brayden-Fletcher-Goldfarb-Shanno (BGFS) algorithm to disturb and assign ligands into the target site, and analyses the scoring function of each ligand conformation (Martiz et al., 2022b; Patil et al., 2022c). Because of the large number of torsions were allowed during ligand formation, ligands were flexible throughout the docking simulation, whereas protein was assumed to be rigid. For ligand molecules, however, 10 degrees of were allowed, out of ten binding poses generated, the first one with zero root-mean square deviation (RMSD) of atomic positions is extremely genuine. It also has the strongest binding affinity of any position, indicating that the binding is more effective. The visualization of molecular docking simulation was completed using Biovia Discovery Studio Visualizer 2021, an open-source visualizing GUI software. The extent of ligand interaction was determined using binding affinity, the total number of intermolecular bonds, and the total number of hydrogen bonds (Patil et al., 2021a; Gurupadaswamy et al., 2022; Shivanna et al., 2022).

#### Molecular dynamics simulation and binding free energy calculations

Proteins with ligands which were chosen along with metformin as standard after docking were selected for the molecular dynamics (MD) simulation. The MD simulation was run using the biomolecular software package of GROMACS-2018.1, according to Patil et al. (2021b) and Belal et al. (2022). GROMACS is a comprehensive software package for performing molecular dynamics or simulating Newtonian equations of motion for systems containing hundreds to millions of particles. It is primarily intended for biological compounds with several complex bound connections, such as proteins, lipids, and nucleic acids. The software is exceptional at computing nonbonded interactions, which are frequently seen as the most important in simulations (Patil et al., 2022a; Martiz et al., 2022c). The CHARMM36 force field (https://www.charmm.org/archive/ charm/resources/charm-force-fields/) (Accessed on 14 February 2023) was used to approximate the ligand structures, and the Swiss Param server (https://www.swissparam.ch/) (Accessed on 14 March 2023) was used to construct the ligand topology. On the other hand, using the pdb2gmx module, protein structure was also added with the CHARMM36 forcefield (Kumar et al., 2021; Patil et al., 2022a; Ganavi et al., 2022). The next step involved 5,000 steps of energy minimization in the vacuum using the steepest descent approach. The distance between each protein complex and the box’s edges was 10 Å. To maintain the necessary 0.15 M salt concentration, the solvent was incorporated into the TIP3P water model with the proper number of Na^+^ and Cl^−^ counterions. In total, 5 simulations were run for 100 ns simulation time at 310 K temperature and 1 bar pressure. The trajectory analysis of root-mean-square deviation (RMSD), root-mean-square-fluctuation (RMSF), the radius of gyration (Rg), solvent accessible-surface-area (SASA), and ligand hydrogen bond parameters was done and the results were plotted in the graphical format using XMGRACE software, a GUI based software used for plotting the results of MD simulation (Patil et al., 2021b; Maradesha et al., 2022; Pushpa et al., 2022).

MD simulation run results for protein-ligand complexes along with standard drug were subjected to binding free energy calculations using the Molecular Mechanics/Poisson-Boltzmann Surface Area (MM-PBSA) technique. It is another application of molecular dynamics simulations and thermodynamics for determining the extent of ligand binding with protein. The g_mmpbsa program with MmPbSaStat.py script, which utilizes the GROMACS 2018.1 trajectories as input, was used to determine the binding free energy for each ligand-protein combination (Patil et al., 2022b; Sajal et al., 2022) in the g_mmpbsa program, three components are used to calculate the binding free energy: molecular mechanical energy, polar and apolar solvation energies, and molecular mechanical energy. The calculation is done using MD trajectories of the last 50 ns were considered to compute ΔG with dt 1,000 frames. It is evaluated using molecular mechanical energy, and polar-a polar solvation energies. Eqs 1, 2 that are used to calculate the free binding energy are given below.

$$\Delta G_{b\mathrm{inding}}=G_{\mathrm{complex}-}\left(G_{\mathrm{protein}}+G_{\mathrm{Ligand}}\right)$$ (1)

$$\Delta G=\Delta E_{\mathrm{MM}}+\Delta G_{\mathrm{Solvation}}\;–\;T\Delta S=\Delta E_{\left(\mathrm{bonded}+\mathrm{non}-\mathrm{bonded}\right)}+\Delta G_{\left(\mathrm{Polar}+\mathrm{non}-\mathrm{polar}\right)}\;–\;T\Delta S$$ (2)

G_Binding_: binding free energy, C_omplex_: total free energy of the protein-ligand complex, G_Protein_ and G_Ligand_: total free energies of the isolated protein and ligand in solvent, respectively, ΔG: standard free energy, ΔE_MM_: average molecular mechanics potential energy in vacuum, G_Solvation_: solvation energy, ΔE: total energy of bonded as well as non-bonded interactions, ΔS: change in entropy of the system upon ligand binding, T: Temperature in Kelvin (Patil et al., 2022a; Patil et al., 2022b).

## Results and discussion

### Preparation of Shivagutika extracts

**FIGURE 1:**
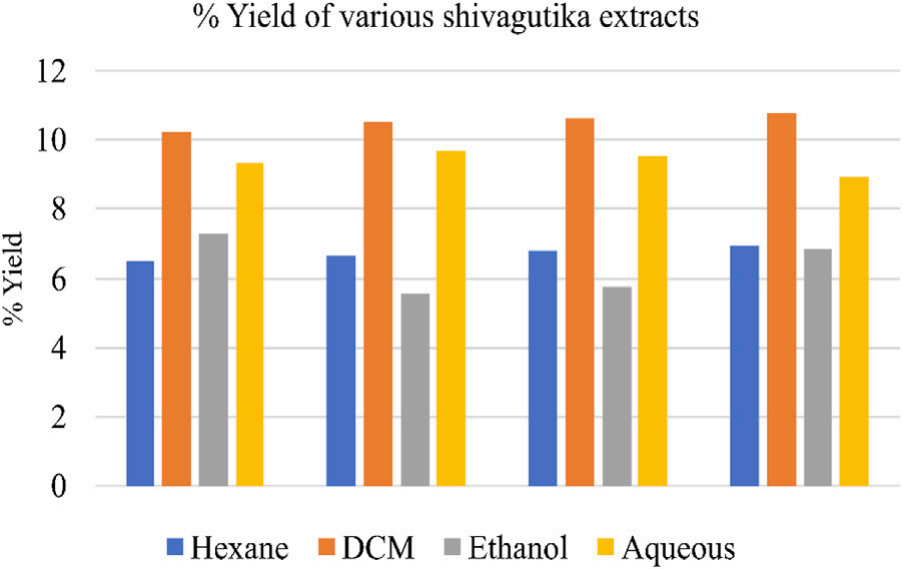
Yield of various Shivagutika extracts.

Weighed 100 g of shivagutika, and subjected to solvent extraction using 4 different solvents n-hexane, dichloromethane (DCM), ethanol and aqueous (water). Percentage yield of total phytochemicals is represented in Figure 1 and Supplementary Table S1. DCM extract exhibited the highest yield for all the 4 batches among all the extracts.

### Estimation of total phenolic acid by Folin—Ciocalteau method

**FIGURE 2:**
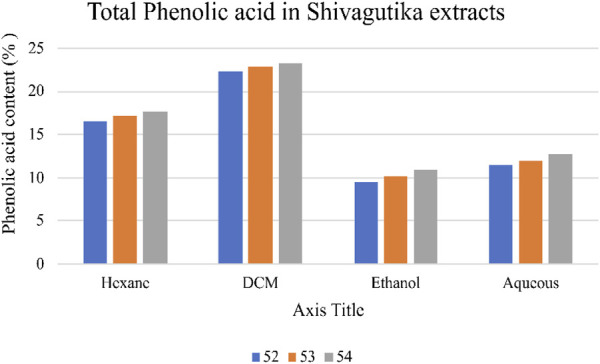
Phenolic acid content of various Shivagutika extracts.

Total phenolic acid content in the Shivagutika extracts was measured using F-C method. DCM extract of possessed higher phenolic content irrespective of 3 batches (52, 53 and 54) and other extracts. After DCM extract, hexane extract exhibited the promising result, followed by aqueous extract and ethanol extract. This could be because DCM has better ability of extraction than other solvents. Similar results were obtained by Kuruburu et al. (2022) in which the phenolic content was 18.00% ± 1.5%. Whereas in the present work, higher phenolic acid of 23.2% was obtained. Phenolic acid content of Shivagutika extracts is represented in Figure 2 and Supplementary Table S2.

### Estimation of total flavonoids by aluminium chloride method

**FIGURE 3:**
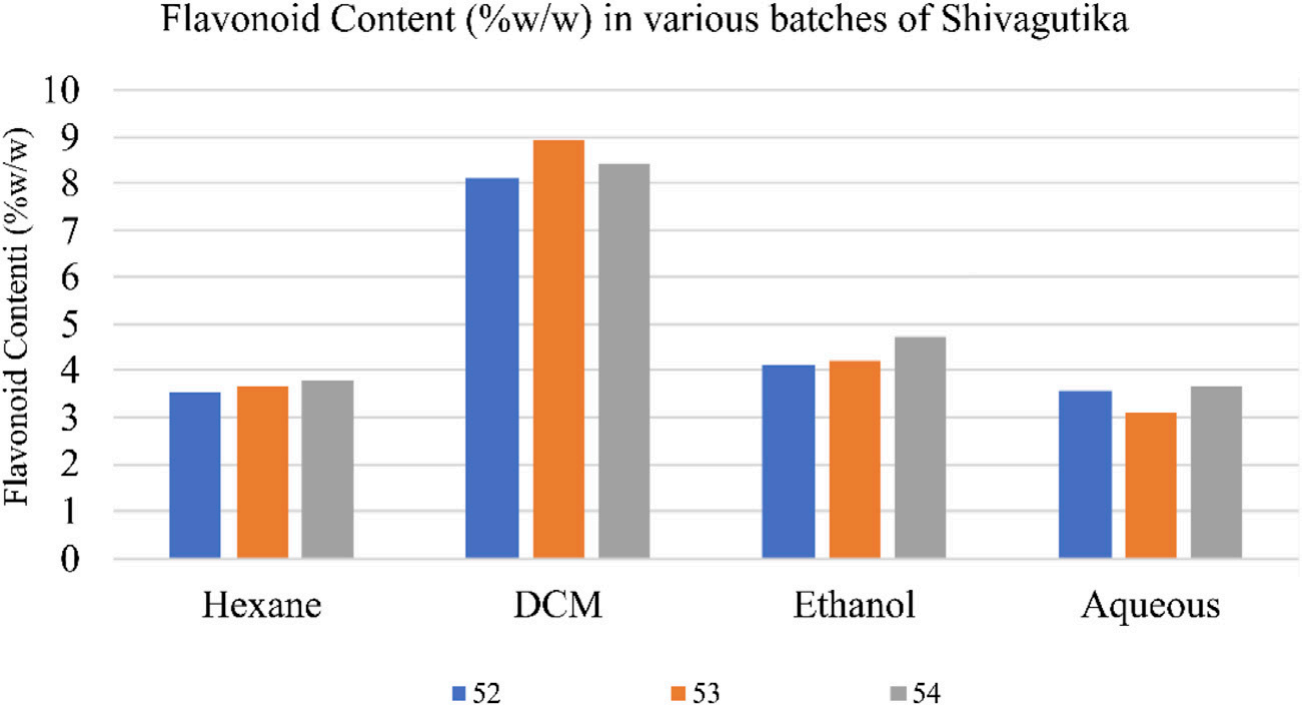
Flavonoids content in shivagutika extracts.

Total flavonoid content in the various Shivagutika extracts was measured using aluminium chloride method. DCM extract of Shivagutika possessed higher flavonoids content irrespective of 3 batches (52, 53, and 54) and other extracts. Total flavonoids content is depicted in Figure 3 and Supplementary Figure S3. The results were in accordance with Vijayalakshmi et al. (2013) wherein the flavonoid concentration was found to be 15.8 mg. Whereas in the present study lower flavonoid content of 8.928 mg was obtained suggesting that Shivagutika possessed lower flavonoid content irrespective of different extracts.

### Estimation of total protein content by Bradford method

**FIGURE 4:**
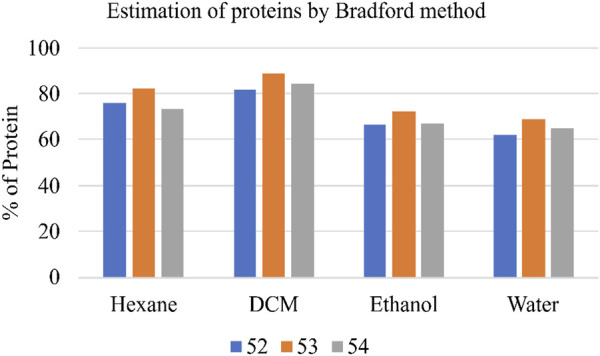
Protein content (%) of various Shivagutika extracts.

Total protein content in the various Shivagutika extracts was measured using Bradford method with BSA as standard. DCM extract of Shivagutika possessed higher protein content irrespective of 3 batches (52, 53, and 54) and other extracts. Protein content of shivagutika extracts is represented in Figure 4 and Supplementary Figure S4. The results were in accordance with the study by Kuruburu et al. (2022) wherein the total protein was 6.3% ± 0.6% Whereas in this study very high protein of 88.92% is obtained in the DCM extract, which suggested Shivagutika contained high protein. Hexane extract showed the second highest protein content followed by the ethanol and aqueous extracts.

### Determination of the antioxidant potential of Shivagutika extracts using ferric reducing antioxidant power (FRAP)

**FIGURE 5:**
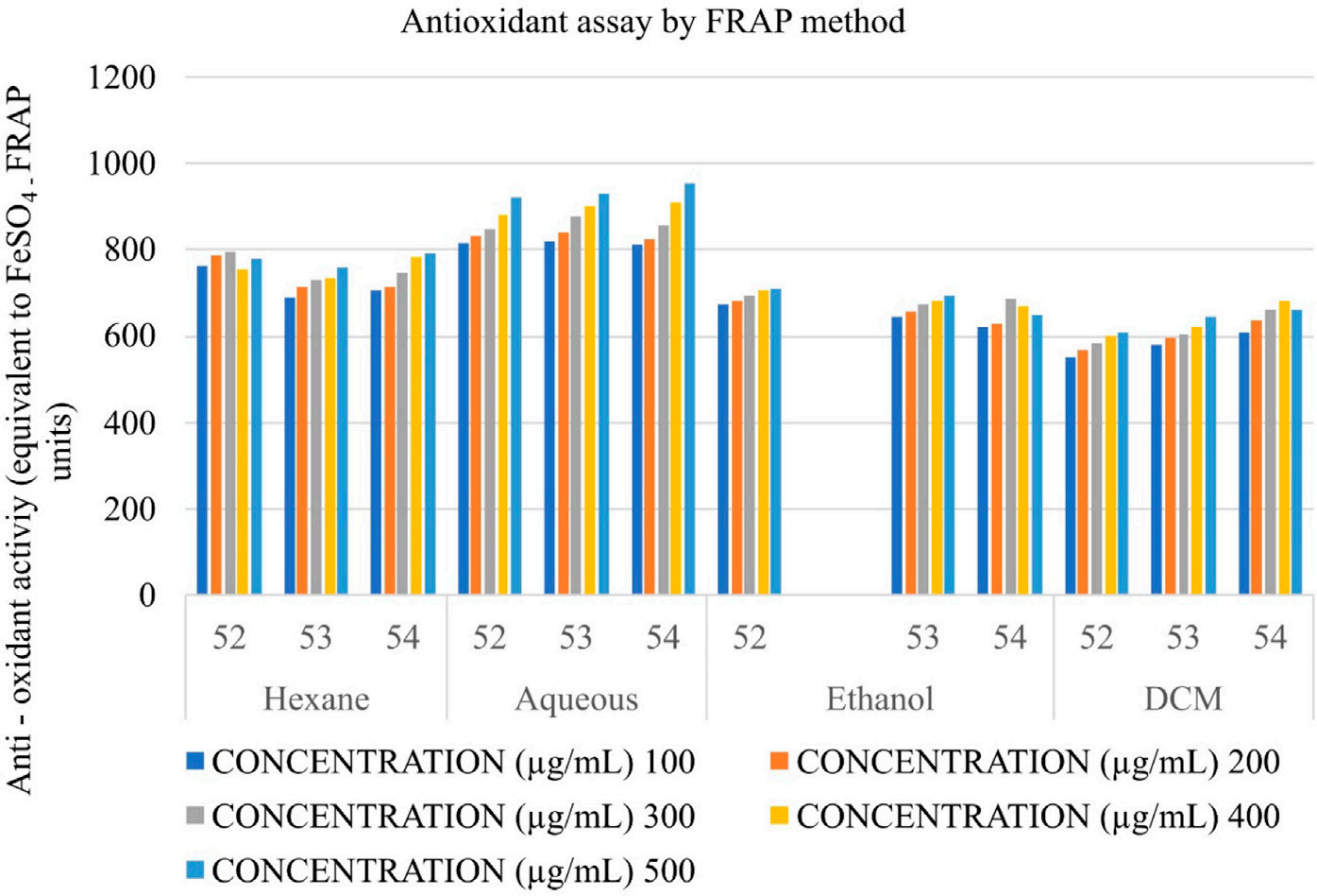
Antioxidant activity of Shivagutika extracts by FRAP assay.

The anti-oxidant potential of Shivagutika extracts was determined using FRAP method with FeSO_4_ as standard. Results showed that DCM extract of Shivagutika exhibited more antioxidant power than other extracts. The results of FRAP assay is represented in and Figure 5 and Supplementary Figure S5. The content and quantity of the phenolic acid components in these extracts may vary, which might explain variances in the antioxidant activity. The results were in accordance with the study by Kuruburu et al. (2022) wherein the dose dependent increase in ferric ion reducing ability of finger millet extract was observed. The present study also suggested the dose dependent increase in the ferric ion reducing ability by the DCM extract. Ethanol extract exhibited the second-best antioxidant activity, followed by hexane and ethanol extract.

### Determination of the antioxidant potential by DPPH (2,2- diphenyl picrylhydrazyl) radical scavenging activity

**FIGURE 6:**
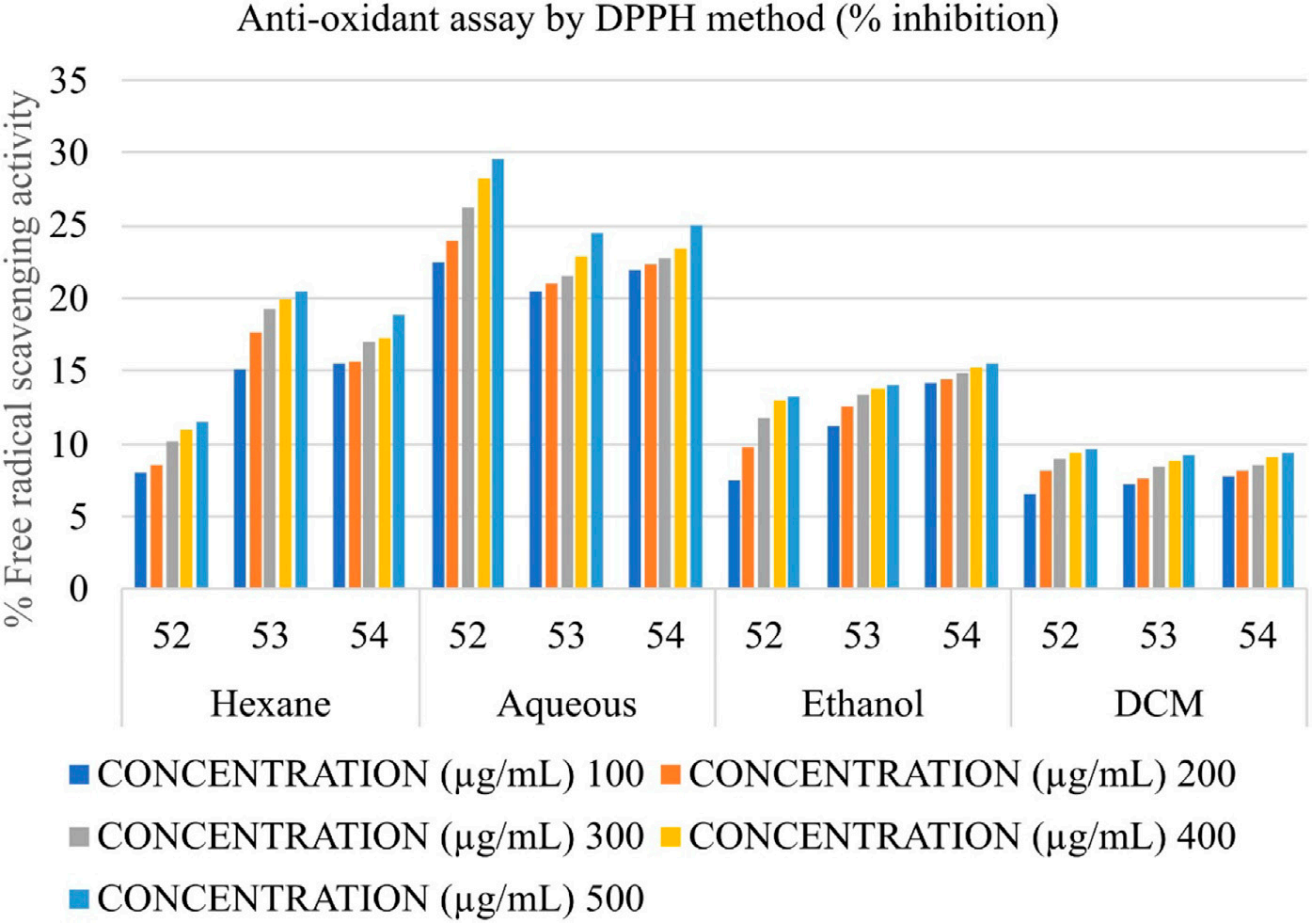
Antioxidant activity of Shivagutika extracts by DPPH method.

The anti-oxidant potential of Shivagutika extracts was determined using DPPH method with Ascorbic acid as standard. Results showed that DCM extract of Shivagutika exhibited more antioxidant power than other extracts. The results of DPPH assay are represented in and Figure 6 and Supplementary Figure S6. The content and quantity of the phenolic acid components in these extracts may vary, which might explain variances in the antioxidant activity. The results were in accordance with the study by Kuruburu et al. (2022) wherein the dose dependent increase in free radical scavenging ability of finger millet extract was observed. The present study also suggested the dose dependent increase in the free radical scavenging activity by the DCM extract. Ethanol extract exhibited the second highest free radical scavenging ability followed by hexane and aqueous extract.

### Identification of phytochemical components of Shivagutika fractions by UPLC—QTOF—MS

**FIGURE 7:**
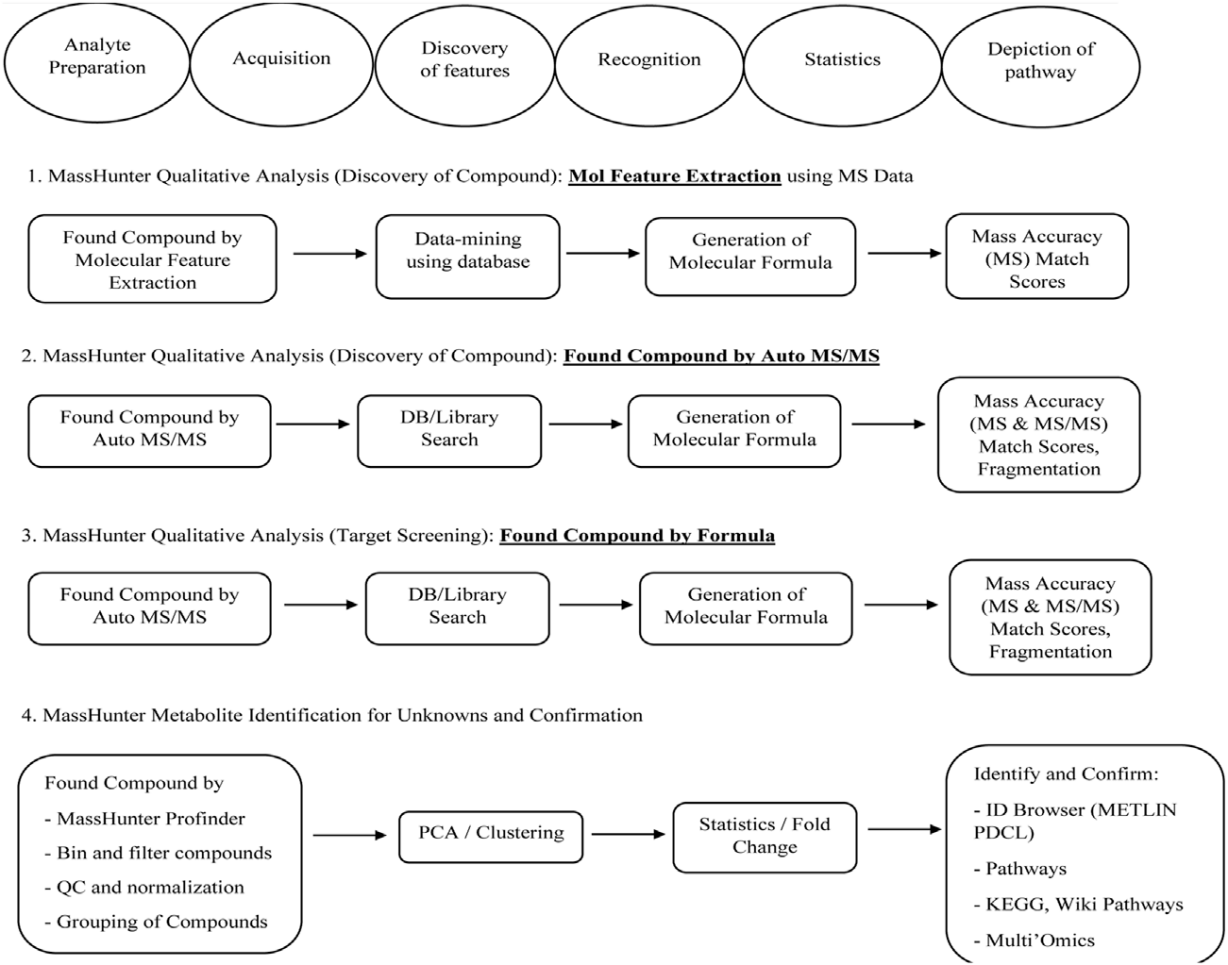
Working diagram of UPLC-QTOF-MS for the identification of compounds present in DCM extracts of Shivagutika.

DCM extract was subjected to UPLC—QTOF—MS analysis as it exhibited highest results with respect to phenolic acid content, protein content, flavonoids content and anti-oxidant activity assay. The phytochemical composition of dichloromethane extracts of different batches was determined by using liquid chromatography coupled with mass spectrometry (LC-MS-MS). Analysis of the data showed the presence of alkaloids, peptides, mono and dicarboxylic acid, Fatty acids, Steroid hormones, Phenols, Flavonoids, Biflavonoids, Conjugate linoleic acids, Ribo disaccharides, Hydroxy isoflavones with varied relative percentages. Working diagram of UPLC-QTOF-MS is represented in Figure 7. Mass spectrograms are represented in Supplementary Figure S1, mass spectrograms of phenolic acids are represented in Supplementary Figure S2. Table 1 represented the LC-MS/MS results of DCM extract. Supplementary Table S7 depicted the phenolic acids of the DCM extract with retention time and Supplementary Table S8 represented the results of LC-MS/MS analysis of Shivagutika DCM extract with percentage. The results were in accordance with Kuruburu et al. (2022) wherein flavonoids, phenolic acids, biflavonoids, flavones and flavans were observed in the finger millet extract. Whereas in this study the LC-MS data showed the presence of alkaloids, peptides, mono and dicarboxylic acid, Fatty acids, Steroid hormones, Phenols, Flavonoids, Biflavonoids, Conjugate linoleic acids, Ribo disaccharides, Hydroxy isoflavones with varied relative percentages.

**TABLE 1:** LC-MS/MS results of Shivagutika DCM extract.

Sr No	Retention Time (min)	Molecular Mass (amu) (experimental)	Molecular Mass (amu) (Theoretical)	IUPAC Name
1	11.53	281.2506	281.251	(2S,3R,4S,5R)-2-[(3R,4R,5R)-4,5,6-trihydroxyoxan-3-yl] oxyoxane-3,4,5-triol (1,4-D-Xylobiose)
2	11.07	279.2346	279.236	(10E,12Z)-octadeca-10,12-dienoic acid (10E, 12Z-octadecadienoic acid)
3	12.06	283.2651	282.254	4-bromo-2,6-ditert-butylphenol (4-Bromo-2,6-di-tert-butylphenol)
4	9.96	579.1311	579.132	5,7-dihydroxy-8-[5-(5-hydroxy-7-methoxy-4-oxochromen-2-yl)-2-methoxyphenyl]-2-(4-methoxyphenyl) chromen-4-one (Sciadopitysin)
5	9.29	295.2287	295.229	(9Z,11E)-13-hydroxyoctadeca-9,11-dienoic acid (13-Hydroxy-9Z,11E-octadecadienoic acid)
6	9.82	295.2288	295.23	icosa-5,8,11,14-tetraynoic acid (5,8,11,14-Eicosatetraynoic acid)
7	10.69	277.2181	277.219	(5Z,9Z,12Z)-octadeca-5,9,12-trienoic acid (Pinolenic acid)
8	7.94	265.1485	265.149	dodecyl hydrogen sulfate (Dodecyl sulfate)
9	14.1	395.3899	395.391	[(2R)-3-hexadecoxy-2-hydroxypropyl] dihydrogen phosphate (1-Hexadecyl lysophosphatidic acid)
10	13.67	367.3587	367.36	[(10R,13S,17S)-10,13-dimethyl-3-oxo-1,2,6,7,8,9,11,12,14,15,16,17-dodecahydrocyclopenta [a]phenanthren-17-yl] hydrogen sulfate (4-Androsten-17. beta. -ol-3-one sulfate)
11	12.66	311.2961	311.297	4-[2-(4-hydroxy-3-propan-2-ylphenyl) propan-2-yl]-2-propan-2-ylphenol 9 (Bisphenol G)
12	9.9	299.2602	299.261	(E)-3-[4-[3-[3,4-dihydroxy-4-(hydroxymethyl) oxolan-2-yl] oxy-4,5-dihydroxy-6-(hydroxymethyl) oxan-2-yl] oxypendyl]-1-(2-hydroxy-4,6-dimethoxyphenyl) prop-2-en-1-one (4,2′-Dihydroxy-4′,6′-dimethoxychalcone)
13	9.04	323.1296	323.13	4-[(3R)-8,8-dimethyl-3,4-dihydro-2H-pyrano [2,3-f] chromen-3-yl] benzene-1,3-diol (Glabridin)

### Determination of anti-proliferative potential

Triple negative breast cancer cell lines were unable to proliferate due to DCM shivagutika extract. MCF-7, MDA MB 231, and MDA MB 468. Using the triple negative breast cancer cell lines MDA-MB-468, MDA-MB-231, and MCF-7 for 48 h each, and counting the number of live cells using the SRB test, DCM Shivagutika extract’s anti-proliferative activity was evaluated. Data analysis exhibited that DCM extract of Shivagutika showed about 70%–80% growth inhibition in MDA-MB- 468 and MCF-7 cell lines at 500 and 1,000 μg/mL concentration. However, DCM shivagutika extracts exhibited a dose dependent growth inhibition pattern only against MDA-MB 468 and MCF-7, but failed to inhibit MB-231 cells. Variations in the efficacy of these fractions against breast cancer could be due to differences in their phenolic acid’s composition or their bioavailability or a mixture of various other factors such as presence of other biomolecules, which might be exhibiting synergistic effect or antagonistic properties. A study by Pant et al. (2012) stated that mineral pitch (Shilajit) induced apoptosis via the production of ROS, and inhibited proliferation of Huh-7 cells by inducing miRNA-22 and inhibiting miRNA-21.49 However, the mechanistic basis of cell growth inhibition is poorly understood. Therefore, further studies are required to identify the key molecules and molecular mechanisms modulated by shivagutika derived phyto compounds. Dichloromethane extracts of Shivagutika (treated for 48 h) had a greater inhibitory impact on MDA MB 468 Breast Cancer Cell Lines than other extracts. The inhibitory impact was compared to vehicle DMSO treated controls and conventional Cisplatin to demonstrate the growth inhibitory effects of Shivagutika fractions.

**FIGURE 8:**
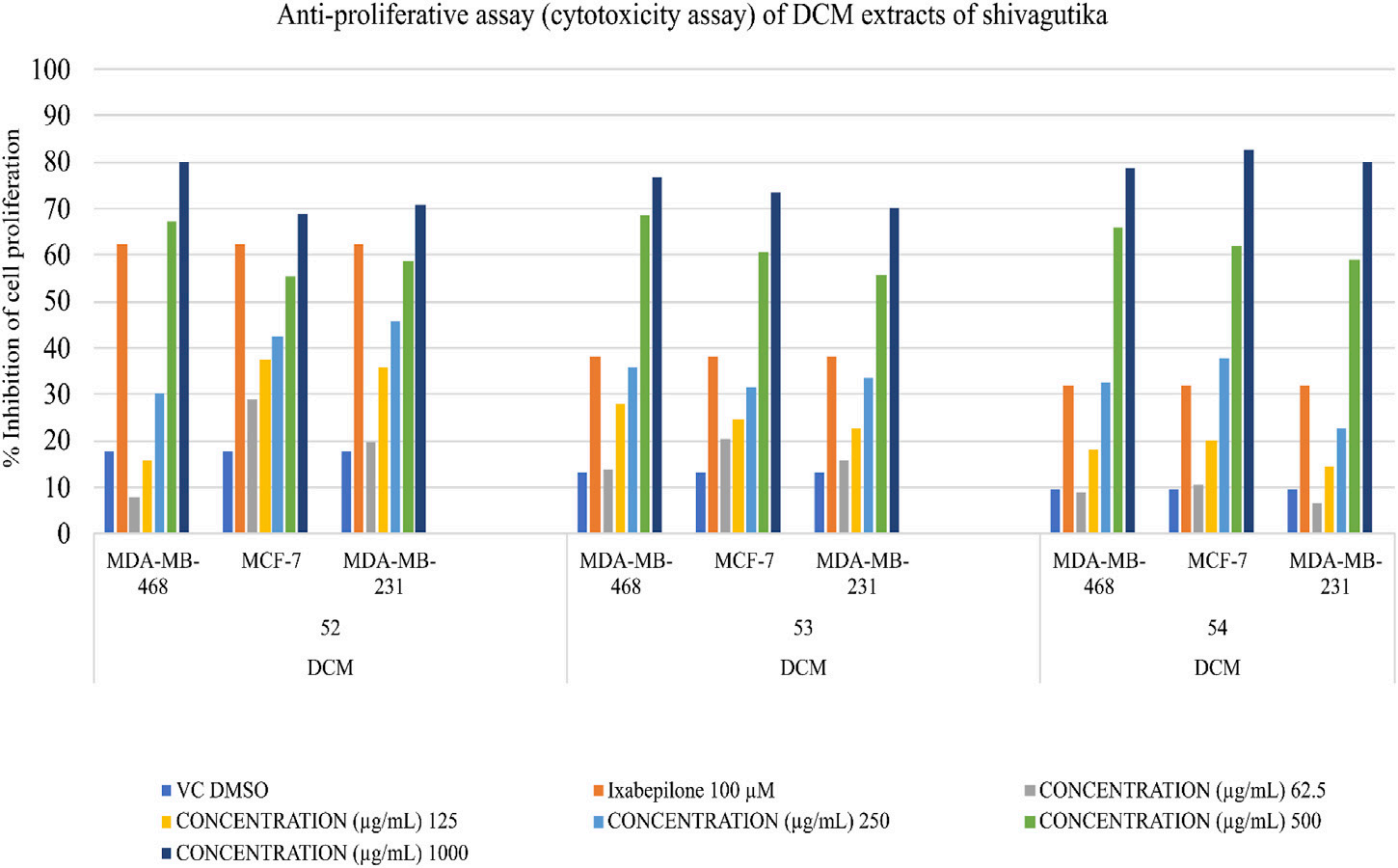
Anti-proliferative (Cytotoxicity) of DCM extracts of Shivagutika.

In comparison to other extracts, Shivagutika extracts in dichloromethane, demonstrated greater inhibitory effects on MDA MB 231 breast cancer cell lines. The growth inhibitory effects of the inhibitor were demonstrated when the inhibitory effect was compared to vehicle-DMSO treated controls and the standard ixabepilone. Results of anti-proliferation (cytotoxicity assay) without IC_50_ values are represented in Figure 8 and Supplementary Table S9 depicted the results of anti-proliferative assay with IC_50_.

As compared to other extracts, Shivagutika extracts in dichloromethane (48 h) had a greater inhibitory impact on the MDA MB 468, MCF 7 and MDA MB 231 breast cancer cell lines. The growth inhibitory effects of Shivagutika fractions were demonstrated by comparing the inhibitory effect (% inhibition) to vehicle DMSO treated controls and conventional ixabepilone.

### Anti-cancer activity of Shivagutika DCM extracts

**FIGURE 9:**
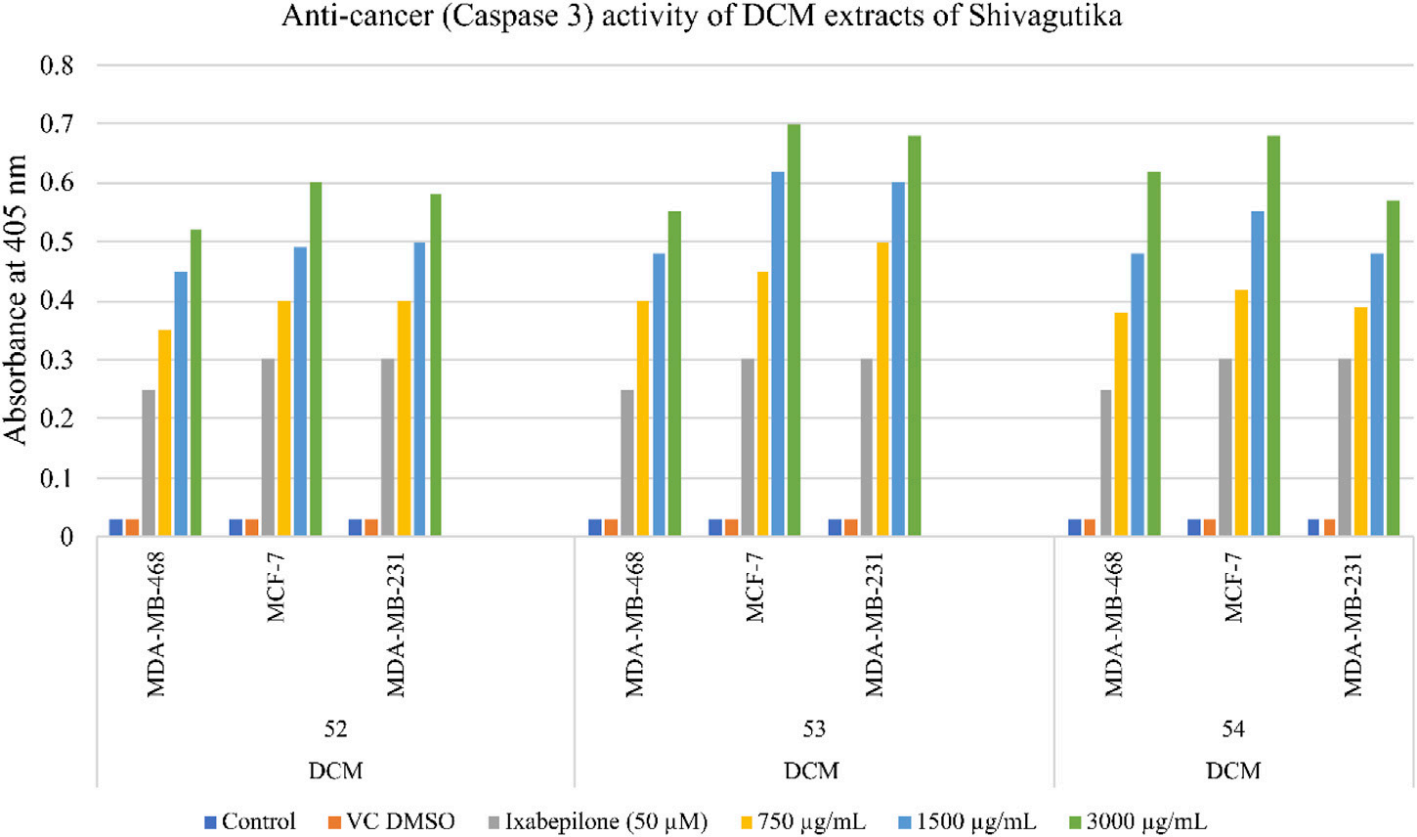
Anti-cancer (Caspase 3) activity of DCM extracts of Shivagutika.

The anti-cancer (mechanism of cell death) activity was performed using Caspase 3/CPP32 colorimetric assay wherein the cell lines were treated with DCM extracts of Shivagutika for 48 hr. Activation of caspase 3 indicated the initiation of apoptosis in mammalian cells. The results of anti-cancer activity are represented in Figure 9 and Supplementary Table S10. DCM extracts of Shivagutika induced cancer cell death by elevating caspase 3 (executionary protease and important mediator of apoptosis). The effect was found to be dose dependent in all cell lines. The results were in accordance with Baharara et al. (2016) wherein the caspase 3 activity increased in cells treated with gold nanoparticles. Similarly, in the present study the caspase 3 activity increased by the DCM extract of Shivagutika in a dose dependent manner.

### Cell death assay with acridine orange (AO) and ethidium bromide (EtBr) staining

**FIGURE 10:**
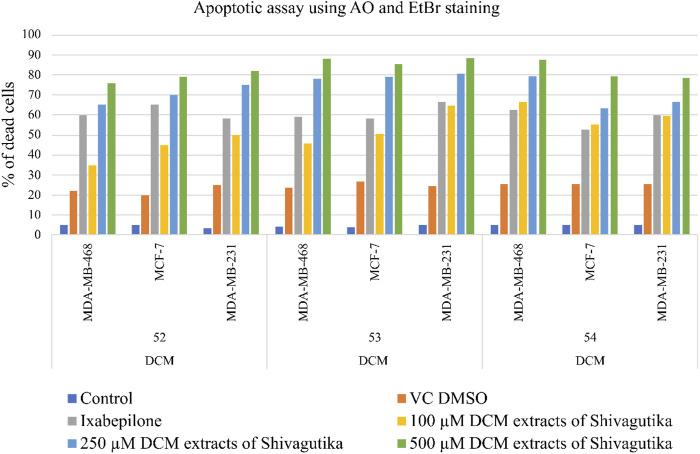
Apoptotic assay using AO and EtBr staining.

**FIGURE 11:**
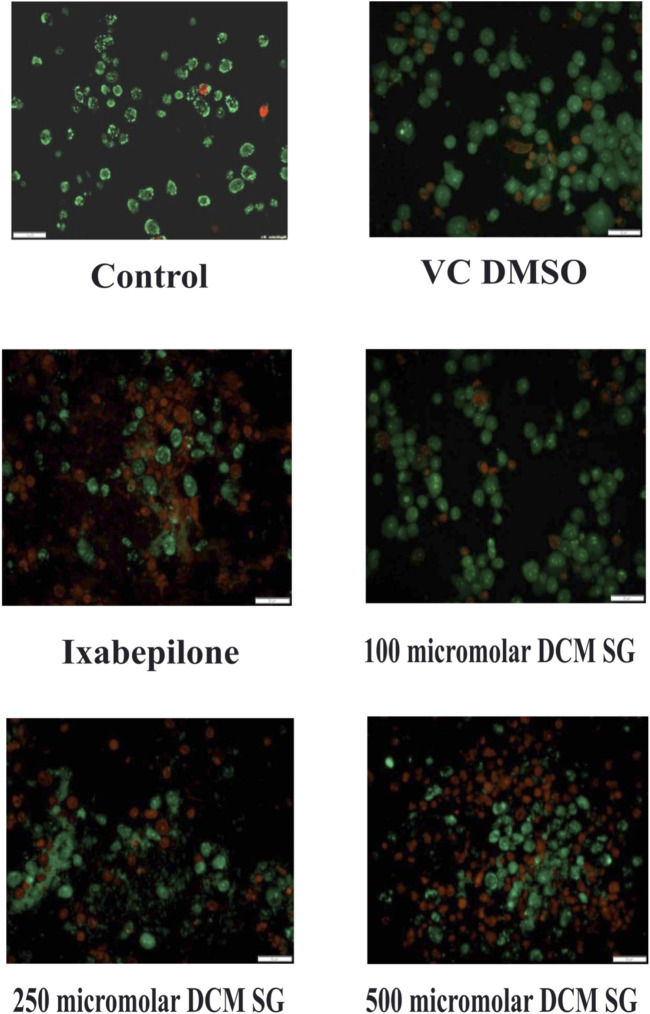
Results of apoptotic assay using AO and EtBr staining.

Acridine orange and ethidium Bromide dual staining is a well reported method to analyze the effect of pharmacological agents on cell death process. Live cells appear green while red cells represent dead ones. DCM extract of Shivagutika treatment induced cell death in breast cancer cell line in a dose dependent manner. Ixabepilone was used as a positive control in this study. Result of cell death (apoptosis assay) is graphically represented in Figures 10, 11 represented the stained images of breast cancer cells. Supplementary Table S11 depicted the percentage of dead cells of apoptotic assay with respect to concentration of the extract.

### Molecular docking simulation

**FIGURE 12:**
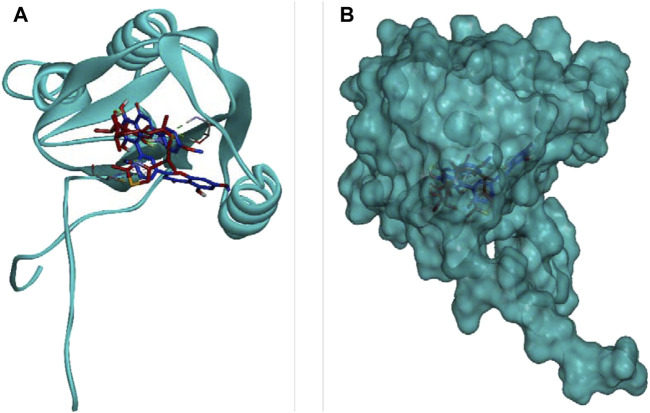
3D representation of Caspase 3 with Sciadopitysin (dark blue) and drug (red) inside the active site **(A)** Ribbon representation and **(B)** Surface representation.

**FIGURE 13:**
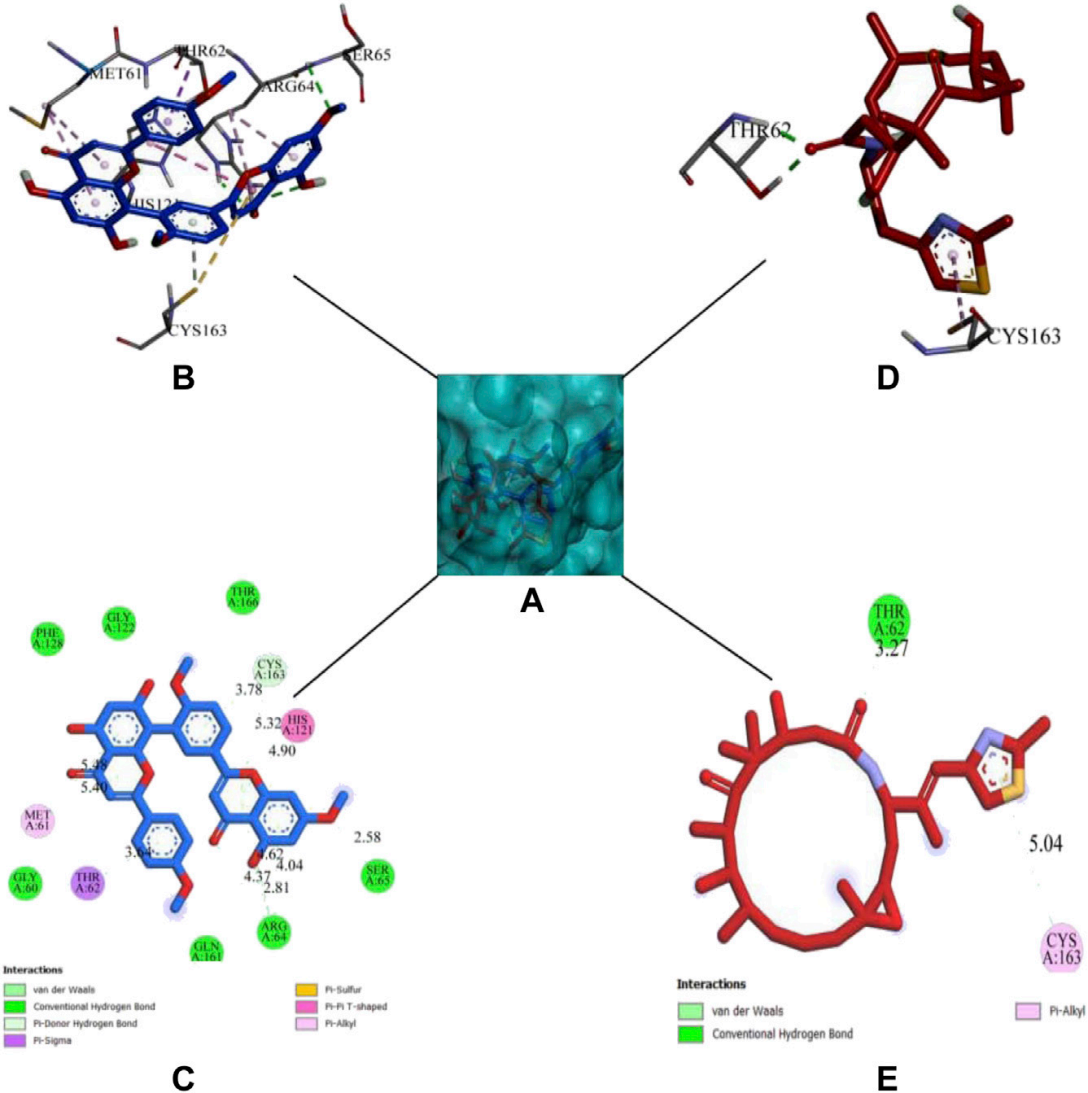
3D representation of Sciadopitysin (blue) and ixabepilone (red) inside the binding cleft of Caspase 3. **(A)** Surface map of Caspase 3 with Sciadopitysin and ixabepilone, **(B,C)**: 3D and 2D representation of Sciadopitysin **(D,E)**: 3D and 2D representation of ixabepilone.

DCM extract of shivagutika was subjected to various analysis, anti-proliferation assay and anti-cancer assay (Caspase 3 activity assay) as it exhibited promising outcomes. Further, the *in silico* analysis (molecular docking) was performed to identify the potent molecule present in the DCM extract responsible for the activation of Caspase 3, protease responsible for apoptosis. The compounds from the LC-MS analysis and standard drug ixabepilone were docked with Caspase 3 as target. Figure 12 depicted the 3D representation of Caspase 3 with Sciadopitysin and standard drug. Figure 13 depicted the 3D and 2D representation of interactions with Caspase 3. The outcomes of molecular docking are represented in Table 2 suggested that Sciadopitysin was found to be the potent molecule since it interacted with Caspase 3 with a binding energy of −7.2 kcal/mol. It formed 12 intermolecular interactions out of which 4 were hydrogen bonds with Arg 64 and Ser 65. This result was in accordance with study performed by Khan et al., 2022 where quercetin isolated from *Phoenix dactylifera* interacted with the same amino acid residues of Caspase 3 as of Sciadopitysin, i. e., Arg64 and Ser65. Whereas ixabepilone formed 3 intermolecular interactions with 2 being hydrogen bonds with binding energy of −5.6 kcal/mol. Table 3 depicted the interactions of Sciadopitysin and ixabepilone.

**TABLE 2:** Results of molecular docking: compounds of DCM extract and ixabepilone with Caspase 3.

Name of the compounds	BA (kcal/mol)	TIH	THB
1,4 D- Xylobiose	−5.4	8	3
1HDLPA	−4.2	7	5
2_(2H_Benzotriazol_2_yl) _4_6_di_tert_pentylphenol	−5.9	9	2
3_4_DMBA	−4.7	10	1
4_2_DH4_6_DM_Chalcone	−5.8	7	3
4_Androsten_17_beta__ol_3_one sulphate	−5.7	6	1
4_bromo_2_6_ditert_butylphenol	−4.3	8	2
5_8_11_14_Eicosatetraynoic acid	−4.4	7	3
5_alpha__Androstane_3_17_dione	−5.9	9	2
10E12ZODDA	−4.5	9	2
13H9Z11EODDA	−4.7	10	3
Arachidonic acid biotinamide	−4.6	11	2
Benzoic acid	−4.0	9	1
Bisphenol G	−5.6	7	2
Caffeic acid	−4.6	6	3
Cinnamic acid	−4.3	5	2
Dodecyl sulphate	−3.8	4	1
Ferulic acid	−4.7	4	2
Glabridin	−5.9	5	1
Hematoporphyrin	−6.0	7	3
Hexadecanoic acid	−3.2	8	1
Pinolenic acid	−4.2	9	2
Piperine	−6.1	4	1
Pristanic acid	−3.9	5	3
Sciadopitysin	**−7.2**	**12**	**4**
Sinapic acid	−5.2	5	1
3_4_5 TMBA	−4.7	6	2
Ixabepilone	**−5.6**	**3**	**2**

Bold values indicates the compound Sciadopitysin with highest binding affinity value and standard drug ixabepilone with less binding affinity value compared to Sciadopitysin.

**TABLE 3:** Binding interactions of Sciadopitysin and ixabepilone with Caspase 3.

Compounds	Hydrogen bonds	Hydrophobic bonds					Other bonds
		Pi—Sigma	Pi-Pi Alkyl	Pi -Pi T shaped	Amide -Pi Stacked	Pi—Alkyl	Pi—Sulphur
Sciadopitysin	Arg64 (2.20 Å)	Thr62 (3.63 Å)	—	His121 (4.90 Å)	—	Met61 (5.40 Å)	Cys163 (3.77 Å)
	Arg64 (2.26 Å)					Met 61 (5.47 Å)	
	Arg64 (2.81 Å)					Arg64 (4.04 Å)	
	Ser65 (2.57 Å)					Arg64 (4.62 Å)	
Ixabepilone	Thr62 (1.84 Å)	—	Cys163 (5.03 Å)	—	—	—	—
	Thr62 (1.96 Å)						

### Molecular dynamics simulations and binding free energy calculations

Docking simulation was validated by molecular dynamic (MD) simulation studies, which explained the dynamic behaviour of the protein-ligand complex with respect to time under a solvated environment. The simulation study provides the analysis of protein-ligand complex RMSD, the Rg, SASA, ligand RMSD, the total number of ligand hydrogen bonds maintained throughout the simulation time, and the variation of secondary structure pattern between the protein and their complexes. The RMSD of the protein-ligand complex depicts the stability of the same throughout the simulation by determining the presence of a ligand inside the binding pocket. The Rg considers the varied masses calculated to root mean square distances considering the central axis of rotation. It considers the capability, shape, and folding during each time step on the whole trajectory throughout the simulation. RMSF concentrates on the protein structural regions that differ the most/least from the mean. SASA measures the area around the hydrophobic core formed between protein-ligand complexes. Further, ligand hydrogen bonds appear during the molecular docking study being analyzed over the total simulation period. All the intermolecular hydrogen bonds between the ligands and the respective protein only were considered during the analysis and plotted accordingly. In this study, 6 simulations were performed at 100 ns time with the Caspase 3 alone and in complex with the representative compounds (Sciadopitysin and ixabepilone).

In case of Caspase 3, both Sciadopitysin and ixabepilone stayed in the inhibitor binding site till the end of the simulation run. The RMSD plot showed that Sciadopitysin increased its stability after 10 ns, whereas the ixabepilone was not stable till 30 ns. The compound 7e was not found with any of the unusual fluctuations in the case of RMSF analysis. This shows that the compound was stable throughout the simulation run. The RMSF pattern of apoprotein and protein—Sciadopitysin complex was similar. Except for the loop region (600 residues) and terminal regions, only minimal fluctuations were observed throughout the simulation of the protein—Sciadopitysin complex. But more fluctuations were found in the case of the protein—ixabepilone complex. In addition, the Rg plot shows that Sciadopitysin was compactly bound to the protein compared to ixabepilone. This binding resulted in a significant decrease in the SASA value of the protein—Sciadopitysin complex. Since the compound efficiently occupied the inhibitor binding site, the available SASA decreased upon the extension of the simulation period. Further, analysis of ligand hydrogen bonds shows that Sciadopitysin formed a maximum number of hydrogen bonds (9) than ixabepilone.

**FIGURE 14:**
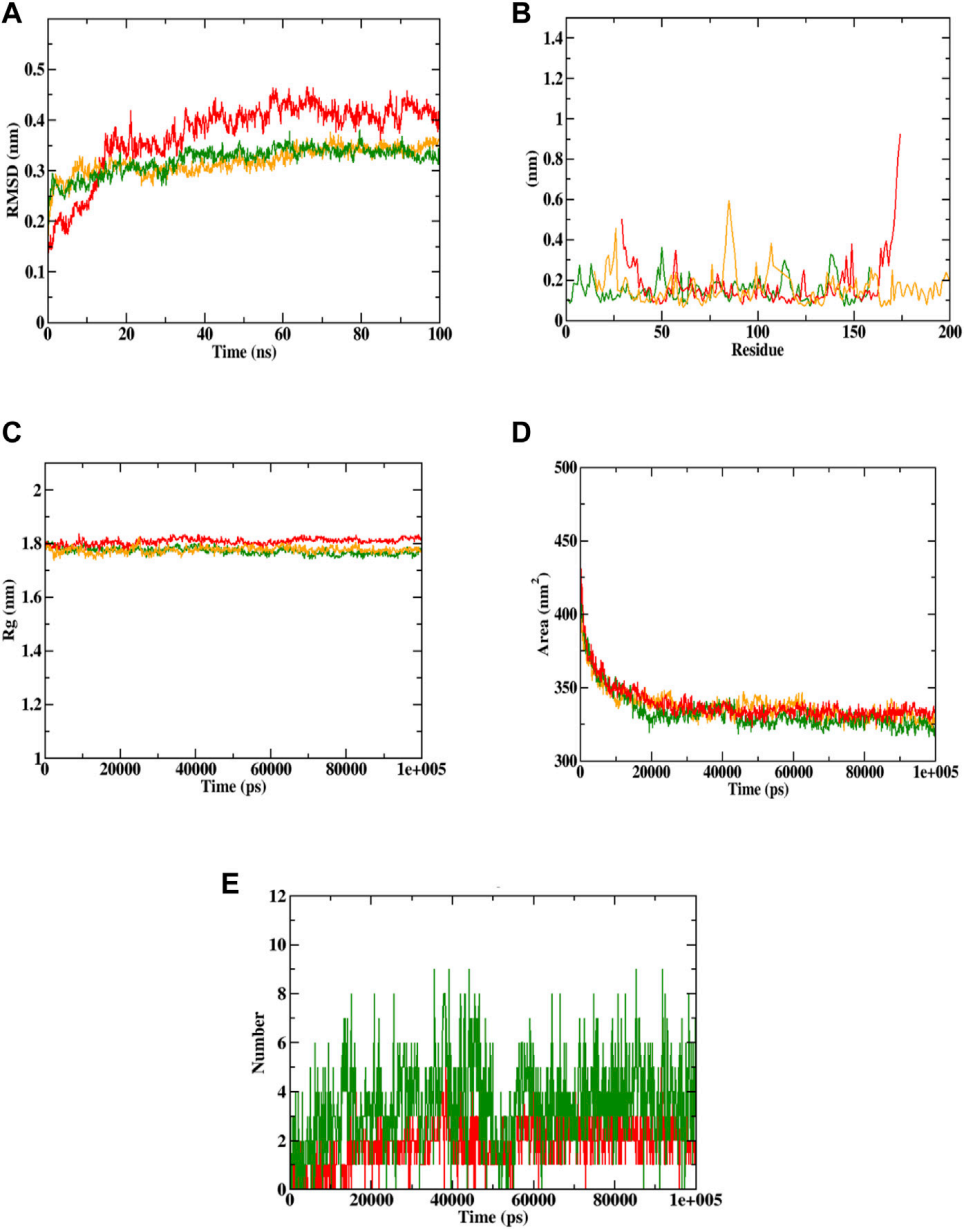
Visualization of MD trajectories of Sciadopitysin and ixabepilone complexed with Caspase 3 run for 100 ns. **(A)** protein-ligand complex RMSD, **(B)** protein-ligand complex RMSF, **(C)** protein-ligand complex Rg, **(D)** protein-ligand complex SASA, **(E)** ligand hydrogen bonds (Red: protein -ixabepilone, green: protein—Sciadopitysin complex, orange: apoprotein).

By the virtue of MD trajectory analysis, both Sciadopitysin and acarbose were stable inside the inhibitor binding site of the Caspase 3 protein. However, trajectory analysis of ixabepilone showed that the compound was comparatively unstable. Although it occupied the same inhibitor binding site as Sciadopitysin, values of trajectory analysis show that the compound is unstable in comparison with Sciadopitysin. Figure 14 represented the trajectory plots obtained from the simulation run. Supplementary Table S12 depicted the MD trajectory values obtained for Caspase 3 apoprotein as well as Caspase 3 protein complexed with Sciadopitysin and ixabepilone and. The results were in accordance with the previous study by Febrina et al. (2021) wherein the MD simulation study was restricted to RMSD and RMSF. Whereas the present study explained all the MD simulation parameters which suggests that this study is accurate compared to the previous study.

Based on the free binding energy calculation it can be predicted that van der Waal’s energy and binding energies had a substantial impact on the complex formation. Based on the energy calculation, the predicted results were mostly energetically favorable. According to the predicted result, the Sciadopitysin bound to Caspase 3 exhibited binding free energy values of −320.515 ± 0.11 kJ/mol. Van der Waal’s binding free energy was shown to be the primary contributor to the formation of complexes when compared to the other energies. Further, when compared to Caspase 3—ixabepilone complex with binding energy of −102.250 ± 0.14, Caspase 3—Sciadopitysin complex were found to have higher (more negative) binding free energies, which indicated that the Caspase 3—ixabepilone complexes have weaker interaction and binding affinity compared to Caspase 3 - Sciadopitysin complex. Supplementary Table S12 depicted the MD trajectory values of Sciadopitysin and ixabepilone complexed with Caspase 3. The study result had a higher pattern compared to previous studies, that have performed binding free energy calculations (Febrina et al., 2021). The values of binding free energies calculated are summarized in Table 4 obtained using the MMPBSA algorithm.

**TABLE 4:** Binding free energy values of target protein complexed with ligands.

Protein-ligand complexes	Types of binding energies				
	Van der Waal’s energy (kJ/mol)	Electrostatic energy (kJ/mol)	Polar solvation energy (kJ/mol)	SASA energy (kJ/mol)	Binding energy (kJ/mol)
Caspase 3—Sciadopitysin	−320.515 ± 0.11	−38.761 ± 0.08	125.530 ± 0.05	−54.768 ± 0.15	−430.991 ± 0.14
Caspase 3—ixabepilone	**−**102.250 ± 0.14	−18.445 ± 0.17	48.350 ± 0.04	−15.329 ± 0.25	−190.705 ± 0.30

The most common malignancy in women that results in mortality is breast cancer (Azamjah et al., 2019). Many experimental investigations, observational studies, meta-analyses, systematic analyses, and epidemiological studies have revealed an adverse relationship between the incidence and death rate of breast tumours and the consumption of diets high in polyphenolic chemicals (Hui et al., 2013; Kapinova et al., 2018).

In this study, the effect of Shiva Gutika’s extracts on Breast cancer cell lines MCF-7, MDA-MB468, and MDA-MB231 have been studied.

In the present study, 4 different extracts of Shivagutika were prepared using different solvents—hexane, dichloromethane (DCM), ethanol and aqueous (water). Similar extracts of Finger millet were prepared to study its effect on breast cancer cell lines—MCF-7, MDA-MB468, and MDA-MB231 (Kuruburu et al., 2022). Total phenolic acid content, total flavonoid content and total protein content were estimated, DCM extract with 3 batches 52, 53, and 54 exhibited the higher values compared to other extracts and it was in accordance with the results of the study by Kuruburu et al. (2022) and Vijayalakshmi et al. (2013). Further, all the extracts were subjected to anti-oxidant assay using FRAP and DPPH method. Yet again, DCM extract exhibited promising results and possessed the anti-oxidant activity in a dose dependent manner and the results were in accordance to the studies conducted by Kuruburu et al. (2022). Due to the promising results by DCM extract, it was subjected to LC-MS analysis which resulted in 20+ compounds of various classes—phenolic acids, flavonoids, fatty acids and many more. Similar compounds were found to be present in the extract of Finger millet (Kuruburu et al., 2022). Further, the DCM extract was subjected to anti-proliferative assay on breast cancer cell lines—MCF-7, MDA-MB468, and MDA-MB231, anti-cancer assay and cell death assay (Note—Ixabepilone was used as positive control in all the *in vitro* assays). It was found that DCM extract exhibited cytotoxic effect and anti—cancer potential by inhibiting cell proliferation, increasing the percentage of dead cells, and activating Caspase 3, thus promoting apoptosis. The results were in accordance with the study by Khan et al. (2022). To identify the potent molecule of the DCM extract, molecular docking simulation was performed with Caspase 3 as target. The compound was found to be Sciadopitysin, a bioflavonoid as it interacted with Caspase 3 with binding energy of −7.2 kcal/mol, highest among all the compounds. Many studies have shown that flavonoids may scavenge free radicals, control cellular metabolism, and protect against illnesses caused by oxidative stress (Gorlach et al., 2015; Chirumbolo et al., 2018; Perez-Vizcaino and Fraga, 2018; Yahfoufi et al., 2018; Abotaleb et al., 2019; Rodríguez-García et al., 2019). Many flavonoids have been shown to have anticancer action, while the exact chemical pathways behind this effect are yet unknown. By inhibiting EGFR/MAPK, PI3K, Akt, and nuclear factor kappa-light-chain-enhancer of activated B cells (NF-B), flavonoids, which function as pro-oxidants, might reduce the proliferation of cancer cells (Abotaleb et al., 2019; Neagu et al., 2019; Rodríguez-García et al., 2019). Dihydromyricetin protects against high glucose-induced endothelial dysfunction in which HIF-1α/ROR2/NF-κB plays an important role (Awad et al., 2022). By virtual screening, it was found that Sciadopitysin interacted with the amino acids of the catalytic site of the Caspase 3 and hence activated it, which proved its anti-cancer potential. Results were like the study conducted by Khan et al. (2022). Molecular dynamics simulation analysis suggested that Caspase 3—Sciadopitysin complex were stable with high binding free energy than Caspase 3—ixabepilone complex. The possible anti-cancer mechanism of Sciadopitysin could be arresting of cell cycle, activation of pro-apoptotic proteins like Bax and executioner caspases such as Caspase 3 (Li et al., 2019). Hence it could be hypothesised that Shivagutika exhibit anti-cancer property.

## Conclusion

The current investigation demonstrated the cytotoxic effect of Shivagutika DCM extract against MCF-7, MDA-MB468, and MDA-MB231 cells. Among the 4 extracts prepared, DCM extract exhibited promising results with respect to yield, phenolic acid content, flavonoid content, and protein content. Therefore, DCM extract was subjected to LC-MS/MS analysis. Results of LC-MS/MS analysis revealed a complex composition with phenolic acids, flavonoids, ribo disaccharides and fatty acids. Further, *in silico* analysis revealed that among all the compounds Sciadopitysin, a biflavonoid, bound to the binding site of Caspase 3 with the highest binding energy of −7.2 kcal/mol by forming 12 intermolecular interactions, out of which 4 were hydrogen bonds. Whereas standard drug ixabepilone bound with Caspase 3 with only 3 intermolecular interactions out of which 2 were hydrogen bonds. Molecular dynamics simulation studies also revealed the strong interaction and stability between Sciadopitysin and Caspase 3 compared to ixabepilone - Caspase 3. The Sciadopitysin formed 9 ligand hydrogen bonds compared to ixabepilone which formed only 5 ligand hydrogen bonds. The synergistic impact of bioactive substances, Sciadopitysin, a bioflavonoid present in the DCM extract of Shivagutika, could be responsible for this activity. As our country is rich in flora, Shivagutika is one among them which has both scientific importance and mythological importance. Hence, the anti-cancer potential of this plant should be studied in detail to determine the bioactive chemicals isolated from Shivagutika and their modes of action *in vitro* and *in vivo* through which we can convey the strength and knowledge of our country towards the field of medicine, Ayurveda which is existing since thousands of years However, Shivagutika’s potential as a future anticancer drug candidate and adjuvant would be more thoroughly evaluated as a result of this research.

## Data Availability

The datasets presented in this study can be found in online repositories. The names of the repository/repositories and accession number(s) can be found in the article.
