# Supplementary material for: Bioactive profiling and evaluation of anti-proliferative and anti-cancerous properties of Shivagutika, an Indian polyherbal formulation synchronizing in vitro and in silico approaches
Source: Front Chem. 2023 May 17;11:1195209. doi: 10.3389/fchem.2023.1195209 (PMC10230648; doi:10.3389/fchem.2023.1195209)
Supplement: Supplementary file 1 [file DataSheet1.docx]

**SUPPLEMENTARY MATERIALS**

**List of Tables**

1. SUPPLEMENTARY TABLE S1. Percentage yield of Shivagutika extracts
2. SUPPLEMENTARY TABLE S2. Phenolic acid content of Shivagutika extracts
3. SUPPLEMENTARY TABLE S3. Total flavonoid content of Shivagutika extracts
4. SUPPLEMENTARY TABLE S4. Protein content of Shivagutika extracts
5. SUPPLEMENTARY TABLE S5. Antioxidant activity of Shivagutika extracts by FRAP assay
6. SUPPLEMENTARY TABLE S6. Antioxidant activity of Shivagutika extracts by DPPH (free radical scavenging) assay
7. SUPPLEMENTARY TABLE S7. Phenolic acids of Shivagutika DCM extract
8. SUPPLEMENTARY TABLE S8. LC-MS/MS result of Shivagutika DCM extract with percentage
9. SUPPLEMENTARY TABLE S9. Anti-proliferative (Cytotoxicity) of DCM extracts of Shivagutika
10. SUPPLEMENTARY TABLE S10. Anti-cancer activity (Caspase 3 activity) of DCM extracts of Shivagutika
11. SUPPLEMENTARY TABLE S11. Results of apoptotic assay using AO and EtBr staining
12. SUPPLEMENTARY TABLE S12. MD trajectory values of Sciadopitysin and ixabepilone complexed with Caspase 3

**List of Figures**

1. SUPPLEMENTARY FIGURE S1. Mass Spectrograms of DCM Shivagutika extract
2. SUPPLEMENTARY FIGURE S2. Mass spectrograms of phenolic acids present in DCM Shivagutika extract with retention time

**Tables**

**SUPPLEMENTARY TABLE S1.** Percentage yield of Shivagutika extracts

| **Extractants** | **52** | **53** | **54** | **57** | **Mean** | **SD** |
| --- | --- | --- | --- | --- | --- | --- |
| Hexane | 6.5 | 6.65 | 6.8 | 6.96 | 6.7275 | 0.1975 |
| DCM | 10.25 | 10.54 | 10.65 | 10.8 | 10.56 | 0.2013 |
| Ethanol | 7.3 | 5.57 | 5.76 | 6.84 | 6.3675 | 0.7241 |
| Aqueous | 9.32 | 9.68 | 9.56 | 8.94 | 9.375 | 0.2824 |

**SUPPLEMENTARY TABLE S2.** Phenolic acid content of Shivagutika extracts

| **Stock concentration of the extract**  **(mg/mL)** | **Extracts of Shivagutika** | **Phenolic acid content (mg/ml) in various batches** | | | **Phenolic acid content**  **(% w/w) in various batches** | | |
| --- | --- | --- | --- | --- | --- | --- | --- |
|  |  | **52** | **53** | **54** | **52** | **53** | **54** |
| 100 mg/mL | Hexane | 16.5 ± 0.21 | 17.2 ± 0.45 | 17.7 ± 0.56 | 16.5 ± 0.21 | 17.2 ± 0.45 | 17.7 ± 0.56 |
|  | DCM | 22.4 ± 1.8 | 22.8 ± 0.85 | 23.2 ± 1.54 | 22.4 ± 1.8 | 22.8 ± 0.85 | 23.2 ± 1.54 |
|  | Ethanol | 9.5 ± 1.8 | 10.2 ± 0.65 | 11 ± 0.84 | 9.5 ± 1.8 | 10.2 ± 0.65 | 11 ± 0.84 |
|  | Aqueous | 11.5 ± 2.5 | 12 ± 3.21 | 12.8 ± 1.4 | 11.5 ± 2.5 | 12 ± 3.21 | 12.8 ± 1.4 |

**SUPPLEMENTARY TABLE S3.** Total flavonoid content of Shivagutika extracts

| **Stock concentration of the extract**  **(mg/mL)** | **Extracts of Shivagutika** | **Flavonoid content (mg/ml) in various batches** | | | **Flavonoid content**  **(% w/w) in various batches** | | |
| --- | --- | --- | --- | --- | --- | --- | --- |
|  |  | **52** | **53** | **54** | **52** | **53** | **54** |
| 100 mg/mL | Hexane | 3.525 ± 0.85 | 3.650 ± 0.66 | 3.789 ± 0.95 | 3.525 ± 0.85 | 3.650 ± 0.66 | 3.789 ± 0.95 |
|  | DCM | 8.105 ± 0.47 | 8.928 ± 1.08 | 8.421 ± 0.56 | 8.105 ± 0.47 | 8.928 ± 1.08 | 8.421 ± 0.56 |
|  | Ethanol | 4.120 ± 0.52 | 4.225 ± 1.03 | 4.701 ± 1.10 | 4.120 ± 0.52 | 4.225 ± 1.03 | 4.701 ± 1.10 |
|  | Aqueous | 3.565 ± 0.23 | 3.120 ± 0.151 | 3.648 ± 0.256 | 3.565 ± 0.23 | 3.120 ± 0.151 | 3.648 ± 0.256 |

**SUPPLEMENTARY TABLE S4.** Protein content of Shivagutika extracts

| **Stock concentration of the extract**  **(mg/mL)** | **Extracts of Shivagutika** | **Protein content (mg/ml) in various batches** | | | **Protein content**  **(%) in various batches** | | |
| --- | --- | --- | --- | --- | --- | --- | --- |
|  |  | **52** | **53** | **54** | **52** | **53** | **54** |
| 100 mg/mL | Hexane | 73.65 ± 0.23 | 82.5 ± 0.52 | 73.28 ± 1.08 | 73.65 ± 0.23 | 82.5 ± 0.52 | 73.28 ± 1.08 |
|  | DCM | 81.58 ± 0.07 | 88.92 ± 0.12 | 81.21 ± 0.32 | 81.58 ± 0.07 | 88.92 ± 0.12 | 81.21 ± 0.32 |
|  | Ethanol | 66.67 ± 0.21 | 72.5 ± 0.32 | 66.7 ± 1.02 | 66.67 ± 0.21 | 72.5 ± 0.32 | 66.7 ± 1.02 |
|  | Aqueous | 62.02 ± 0.97 | 69.07 ± 0.59 | 64.42 ± 2.65 | 62.02 ± 0.97 | 69.07 ± 0.59 | 64.42 ± 2.65 |

**SUPPLEMENTARY TABLE S5.** Antioxidant activity of Shivagutika extracts by FRAP assay

| **EXTRACTS** | **BATCHES** | **CONCENTRATION (µg/mL)** | | | | |
| --- | --- | --- | --- | --- | --- | --- |
|  |  | 100 | 200 | 300 | 400 | 500 |
| Hexane | 52 | 763.550 ± 0.12 | 785.450 ± 1.10 | 795.396 ± 2.03 | 756.685 ± 10.20 | 780.650 ± 9.50 |
|  | 53 | 690.150 ± 11.20 | 715.180 ± 12.00 | 729.260 ± 5.00 | 735.915 ± 6.50 | 760.000 ± 18.20 |
|  | 54 | 706.10 ± 15.20 | 715.570 ± 19.50 | 745.150 ± 21.20 | 784.240 ± 32.00 | 790.480 ± 15.00 |
| Aqueous | 52 | 814.500 ± 1.12 | 830.154 ± 1.50 | 850.130 ± 5.20 | 879.930 ± 7.20 | 920.350 ± 10.00 |
|  | 53 | 820.300 ± 08.50 | 840.200 ± 7.55 | 875.165 ± 6.45 | 900.450 ± 12.20 | 930.650 ± 15.00 |
|  | 54 | 810.150 ± 10.20 | 822.334 ± 12.52 | 854.630 ± 13.55 | 910.210 ± 14.40 | 955.230 ± 10.09 |
| Ethanol | 52 | 675.120 ± 06.50 | 680.135 ± 7.02 | 695.180 ± 5.15 | 705.155 ± 11.45 | 710.240 ± 9.65 |
|  | 53 | 645.150 ± 20.20 | 659.184 ± 15.20 | 672.164 ± 13.22 | 680.168 ± 14.66 | 695.154 ± 18.66 |
|  | 54 | 622.120 ± 17.48 | 630.157 ± 16.95 | 684.450 ± 14.23 | 667.610 ± 17.56 | 650.665 ± 20.00 |
| DCM | 52 | 550.150 ± 15.50 | 568.120 ± 18.65 | 584.195 ± 25.20 | 600.157 ± 22.20 | 610.165 ± 19.32 |
|  | 53 | 580.184 ± 17.00 | 595.189 ± 18.86 | 605.100 ± 19.35 | 620.154 ± 21.00 | 645.230 ± 22.00 |
|  | 54 | 610.120 ± 12.50 | 635.154 ± 15.00 | 660.159 ± 16.50 | 679.950 ± 14.70 | 660.194 ± 17.70 |

**SUPPLEMENTARY TABLE S6.** Antioxidant activity of Shivagutika extracts by DPPH (free radical scavenging) assay

| **EXTRACTS** | **BATCHES** | **CONCENTRATION (µg/mL)** | | | | |
| --- | --- | --- | --- | --- | --- | --- |
|  |  | **100** | **200** | **300** | **400** | **500** |
| Hexane | 52 | 8.0 ± 0.21 | 8.5 ± 0.25 | 10.1 ± 0.15 | 11.0 ± 0.10 | 11.5 ± 0.04 |
|  | 53 | 15.1 ± 0.11 | 17.7 ± 0.22 | 19.3 ± 0.35 | 19.9 ± 1.10 | 20.5 ± 0.95 |
|  | 54 | 15.5 ± 1.50 | 15.7 ± 1.54 | 17.0 ± 1.65 | 17.2 ± 1.44 | 18.8 ± 1.15 |
| Aqueous | 52 | 22.5 ± 0.21 | 24.0 ± 0.29 | 26.2 ± 0.31 | 28.2 ± 0.42 | 29.5 ± 0.45 |
|  | 53 | 20.5 ± 0.31 | 21.0 ± 0.36 | 21.5 ± 0.38 | 22.9 ± 0.4 | 24.5 ± 0.44 |
|  | 54 | 22.0 ± 0.51 | 22.3 ± 0.56 | 22.7 ± 0.63 | 23.4 ± 0.69 | 25.0 ± 0.48 |
| Ethanol | 52 | 7.5 ± 0.02 | 9.8 ± 0.09 | 11.8 ± 0.15 | 13.0 ± 0.52 | 13.2 ± 0.65 |
|  | 53 | 11.2 ± 0.41 | 12.6 ± 1.10 | 13.3 ± 0.95 | 13.7 ± 0.68 | 14.0 ± 0.69 |
|  | 54 | 14.2 ± 1.42 | 14.5 ± 1.54 | 14.8 ± 1.65 | 15.2 ± 1.52 | 15.5 ± 1.70 |
| DCM | 52 | 6.5 ± 0.50 | 8.2 ± 0.23 | 8.9 ± 0.42 | 9.3 ± 0.51 | 9.6 ± 1.0 |
|  | 53 | 7.2 ± 1.2 | 7.6 ± 1.05 | 8.4 ± 1.90 | 8.8 ± 1.02 | 9.2 ± 1.25 |
|  | 54 | 7.8 ± 0.65 | 8.2 ± 0.78 | 8.6 ± 0.47 | 9.1 ± 0.13 | 9.4 ± 1.00 |

**SUPPLEMENTARY TABLE S7.** Phenolic acids of Shivagutika DCM extract

| **Sr. no** | **Name of the Phenolic acid** | **Retention time**  **(minutes)** |
| --- | --- | --- |
| 1 | Caffeic acid | 16.474 |
| 2 | Sinapic acid | 21.724 |
| 3 | Ferulic acid | 22.166 |
| 4 | 3.4. DMBA | 22.851 |
| 5 | 3.4.5 TMBA | 25.724 |
| 6 | Benzoic acid | 25.978 |
| 7 | Cinnamic acid | 33.071 |

**SUPPLEMENTARY TABLE S8.** LC-MS/MS result of Shivagutika DCM extract with percentage

| Piperine 78.01% | Pristanic acid (1.89%) |
| --- | --- |
| Hematoporphyrin (5.38%) | Phe-Arg (1.50%) |
| Trp-Trp-Arg (3.87%**)** | Hexadecanoic acid (1.32%) |
| Trp-Tyr-Arg (3.35%) | Arachidonic acid biotinamide P 5(1.10%) |
| 5.alpha. -Androstane-3,17-dione (2.64%) | 2-(2H-Benzotriazol-2-yl)-4,6-di-tert-pentylphenol (0.91%) |
| 1,4-D-Xylobiose (39.81%) | Asiatic acid |
| 10E,12Z-octadecadienoic acid (29.13%) | 3-Cysteinylacetaminophen |
| 4-Bromo-2,6-di-tert-butylphenol (10.96%) | Bisphenol G |
| Guanosine | N-Oleoyl-L-serine |
| 5,8,11,14-Eicosatetraynoic acid (4.73%) | 4,2'-Dihydroxy-4',6'-dimethoxychalcone |
| Glabridin | 8,11-Eicosadiynoic acid |
| Pinolenic acid (3.66%) | 4-Androsten-17. beta. -ol-3-one sulphate |
| Sciadopitysin (3.20%) | Arachidonic sulfonic acid |
| 5,3'-Dihydroxyflavone | 1-Hexadecyl lysophosphatidic acid |
| Dodecyl sulphate (1.93%) |  |

**SUPPLEMENTARY TABLE S9.** Anti-proliferative (Cytotoxicity) of DCM extracts of Shivagutika

| **EXTRACTS** | **BATCHES** | **Cell Lines** | **CONCENTRATION (µg/mL) of DCM extracts with % inhibition and IC_50_** | | | | | | | |
| --- | --- | --- | --- | --- | --- | --- | --- | --- | --- | --- |
|  |  |  | **VC DMSO** | **Ixabepilone** | **62.5** | **125** | **250** | **500** | **1000** | **IC_50_** |
| DCM | 52 | MDA-MB-468 | 17.575 | 62.120 | 7.7260 | 15.7521 | 30.2550 | 67.1850 | 80.0500 | 345.65 |
|  |  | MCF-7 | 17.575 | 62.120 | 28.8550 | 37.6550 | 42.6000 | 55.1500 | 68.8002 | 530.81 |
|  |  | MDA-MB-231 | 17.575 | 62.120 | 19.85 | 35.75 | 45.84 | 58.67 | 70.850 | 443 |
| SD | - | - | 0 | 0 | 5.50 | 14.20 | 6.85 | 7.362 | 5.755 | - |
| DCM | 53 | MDA-MB-468 | 12.976501 | 38.01567 | 13.8550 | 27.798 | 35.950 | 68.652 | 76.6400 | 352.60 |
|  |  | MCF-7 | 12.976501 | 38.01567 | 20.250 | 24.520 | 31.650 | 60.440 | 73.541 | 392.29 |
|  |  | MDA-MB-231 | 12.976501 | 38.01567 | 15.85 | 22.570 | 33.450 | 55.552 | 70.251 | 390.35 |
| SD | - | - | 0 | 0 | 4.81 | 12.54 | 7.65 | 6.89 | 6.20 | - |
| DCM | 54 | MDA-MB-468 | 9.3954 | 32.0165 | 8.925 | 17.920 | 32.561 | 65.982 | 78.653 | 343.05 |
|  |  | MCF-7 | 9.3954 | 32.0165 | 10.500 | 19.899 | 37.884 | 62.021 | 82.562 | 392.35 |
|  |  | MDA-MB-231 | 9.3954 | 32.0165 | 6.566 | 14.565 | 22.520 | 59.020 | 80.200 | 420.93 |
| SD | - | - | 0 | 0 | 5.22 | 8.964 | 7.002 | 12.005 | 9.555 | - |

**SUPPLEMENTARY TABLE S10.** Anti-cancer activity (Caspase 3 activity) of DCM extracts of Shivagutika

| **Extracts** | **Batches** | **Breast Cancer Cell lines** | **Absorbance at 405 nm with concentration of the extract** | | | | | |
| --- | --- | --- | --- | --- | --- | --- | --- | --- |
|  |  |  | **Control** | **VC -DMSO** | **Ixabepilone (50 µM)** | **750 µg / mL** | **1500 µg / mL** | **3000 µg / mL** |
| DCM | 52 | MDA-MB-468 | 0.03 | 0.03 | 0.25 | 0.35 | 0.45 | 0.52 |
|  |  | MCF-7 | 0.03 | 0.03 | 0.3 | 0.4 | 0.49 | 0.6 |
|  |  | MDA-MB-231 | 0.03 | 0.03 | 0.3 | 0.4 | 0.5 | 0.58 |
| DCM | 53 | MDA-MB-468 | 0.03 | 0.03 | 0.25 | 0.4 | 0.48 | 0.55 |
|  |  | MCF-7 | 0.03 | 0.03 | 0.3 | 0.45 | 0.62 | 0.7 |
|  |  | MDA-MB-231 | 0.03 | 0.03 | 0.3 | 0.5 | 0.6 | 0.68 |
| DCM | 54 | MDA-MB-468 | 0.03 | 0.03 | 0.25 | 0.38 | 0.48 | 0.62 |
|  |  | MCF-7 | 0.03 | 0.03 | 0.3 | 0.42 | 0.55 | 0.68 |
|  |  | MDA-MB-231 | 0.03 | 0.03 | 0.3 | 0.39 | 0.48 | 0.57 |

**SUPPLEMENTARY TABLE S11.**  Results of apoptotic assay using AO and EtBr staining

| **Extract** | **Batches** | **Breast cancer cell lines** | **Percentage of dead cells with concentration of the extract** | | | | | |
| --- | --- | --- | --- | --- | --- | --- | --- | --- |
|  |  |  | **Control** | **VC -DMSO** | **Ixabepilone** | **100 µM** | **250 µM** | **500 µM** |
| DCM | 52 | MDA-MB-468 | 5 | 22 | 60 | 35 | 65 | 76 |
|  | | MCF-7 | 5 | 20 | 65 | 45 | 70 | 79 |
|  |  | MDA-MB-231 | 3.5 | 25 | 58 | 50 | 75 | 82 |
| DCM | 53 | MDA-MB-468 | 4.5 | 23.5 | 59 | 45.5 | 78 | 88 |
|  | | MCF-7 | 4 | 26.5 | 58 | 50.5 | 78.9 | 85.5 |
|  |  | MDA-MB-231 | 5 | 24.5 | 66.5 | 64.5 | 80.5 | 88.5 |
| DCM | 54 | MDA-MB-468 | 5 | 25.5 | 62.5 | 66.5 | 79.5 | 87.45 |
|  | | MCF-7 | 5 | 25.5 | 52.5 | 55 | 63.45 | 79.5 |
|  |  | MDA-MB-231 | 5 | 25.5 | 60 | 59.5 | 66.45 | 78.5 |

**SUPPLEMENTARY TABLE S12.** MD trajectory values of Sciadopitysin and ixabepilone complexed with Caspase 3

| **MD Trajectories** | **Apoprotein** | **Protein - Sciadopitysin complex** | **Protein – ixabepilone complex** |
| --- | --- | --- | --- |
| RMSD (nm) | 0.30 | 0.30 | 0.35 |
| RMSF (nm) | 0.59 | 0.31 | 0.90 |
| Rg (nm) | 1.78 | 1.78 | 1.80 |
| SASA (nm^2^) | 325 | 324 | 325 |
| Ligand H-bonds (max.) | **-** | 9 | 5 |

**Figures**


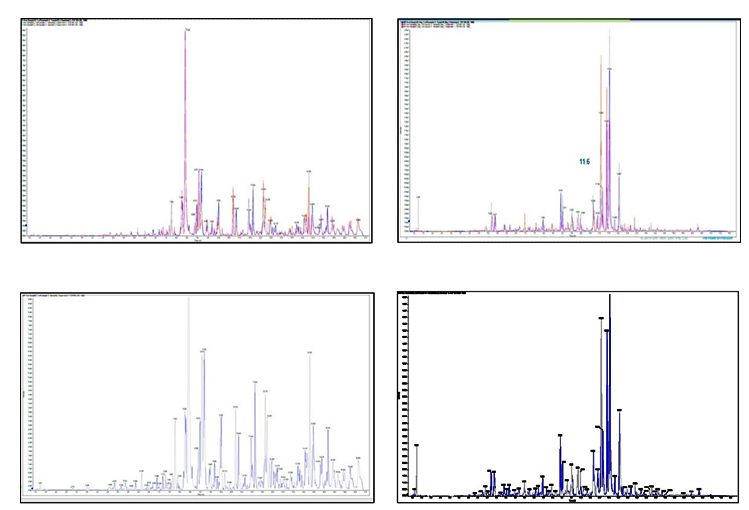


**SUPPLEMENTARY FIGURE S1**. Mass Spectrograms of DCM Shivagutika extract


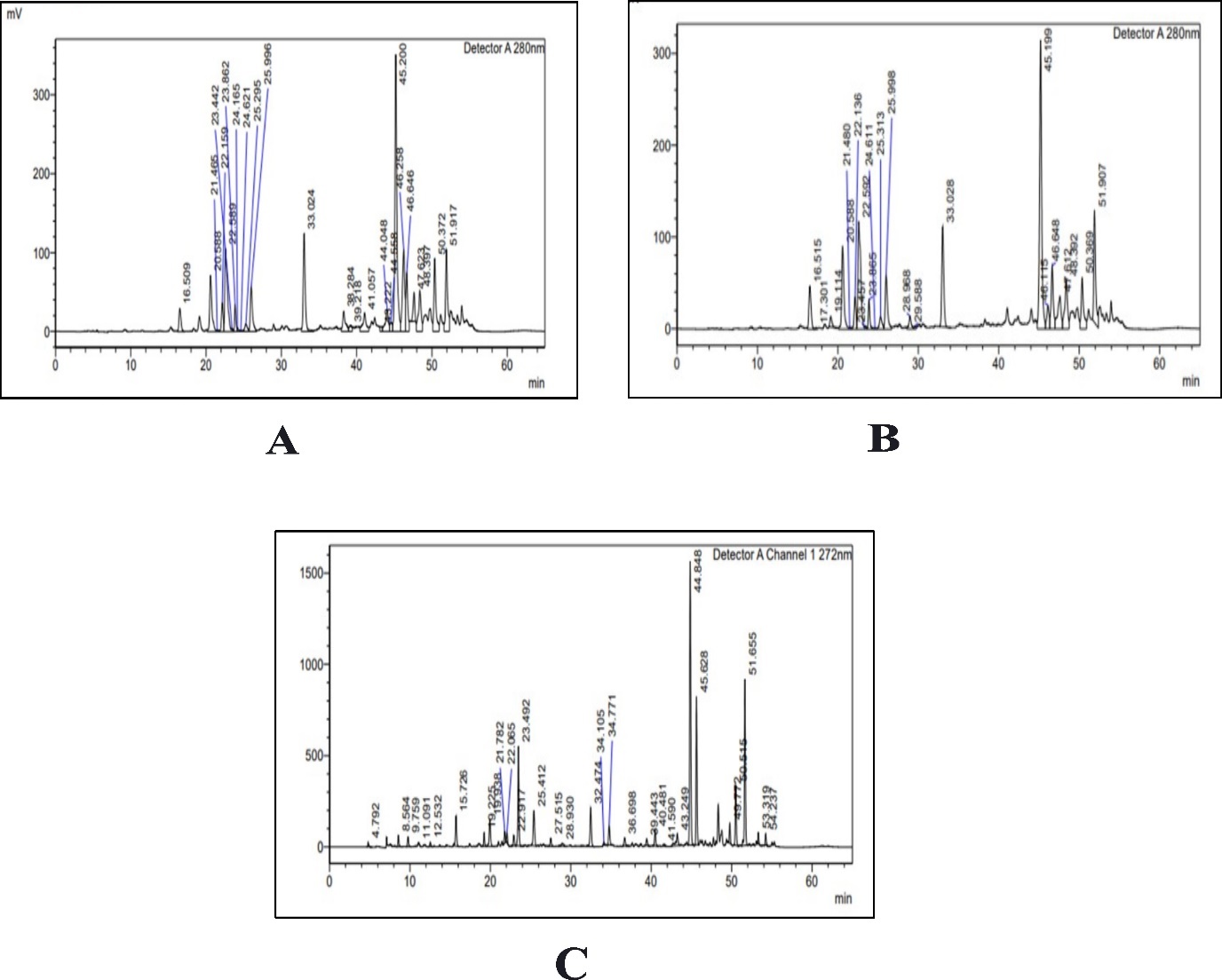


**SUPPLEMENTARY FIGURE S2.** Mass spectrograms of phenolic acids present in DCM Shivagutika extract with retention time 16.509 - Caffeic acid, 21.465- Sinapic acid, 22.159- Ferulic acid, 22.589-3,4 DMBA, 25.295- 3,4,5 TMBA, 25.996- Benzoic acid ,33.024- Cinnamic acid (**A**) 16.515 –Caffeic acid, 21.48- Sinapic acid, 22.136- ferulic acid, 22.592- 3,4 DMBA, 25.313 - 3,4,5 TMBA, 25.998- Benzoic acid, 28.968-3,4, DMCA, 33.028- Cinnamic acid (**B**) 4.792 –Vanillic acid, 8.564- Gallic acid, 11.091-3,4 DHBA, 15.726- 4, hydroxy benzoic acid, 21.782- coumaric acid, 22.065- Ferulic acid, 22.917- 3,4 DMBA, 25.412- 3,4,5-TMBA, 28.93- 3,4 DMCA, 32.474 – Cinnamic acid (**C**).
